# Distinctive variation in the U3R region of the 5' Long Terminal Repeat from diverse HIV-1 strains

**DOI:** 10.1371/journal.pone.0195661

**Published:** 2018-04-17

**Authors:** Christelle Mbondji-wonje, Ming Dong, Xue Wang, Jiangqin Zhao, Viswanath Ragupathy, Ana M. Sanchez, Thomas N. Denny, Indira Hewlett

**Affiliations:** 1 Laboratory of Molecular Virology, Division of Emerging and Transfusion Transmitted Diseases, Center for Biologics Evaluation and Research, Food and Drug Administration, Silver Spring, Maryland, United States of America; 2 Department of Molecular Biology, Faculty of Medicine, Pharmacy and Biomedical sciences, University of Douala, Douala, Cameroon; 3 U.S. Military HIV Research Program, Silver Spring, Maryland United States of America; 4 Department of Medicine, Duke Human Vaccine Institute, Duke University Medical Center, Durham, North Carolina, United States; Fudan University, CHINA

## Abstract

Functional mapping of the 5’LTR has shown that the U3 and the R regions (U3R) contain a cluster of regulatory elements involved in the control of HIV-1 transcription and expression. As the HIV-1 genome is characterized by extensive variability, here we aimed to describe mutations in the U3R from various HIV-1 clades and CRFs in order to highlight strain specific differences that may impact the biological properties of diverse HIV-1 strains. To achieve our purpose, the U3R sequence of plasma derived virus belonging to different clades (A1, B, C, D, F2) and recombinants (CRF02_AG, CRF01_AE and CRF22_01A1) was obtained using Illumina technology. Overall, the R region was very well conserved among and across different strains, while in the U3 region the average inter-strains nucleotide dissimilarity was up to 25%. The TAR hairpin displayed a strain-distinctive cluster of mutations affecting the bulge and the loop, but mostly the stem. Like in previous studies we found a TATAA motif in U3 promoter region from the majority of HIV-1 strains and a TAAAA motif in CRF01_AE; but also in LTRs from CRF22_01A1 isolates. Although LTRs from CRF22_01A1 specimens were assigned CRF01_AE, they contained two NF-kB sites instead of the single TFBS described in CRF01_AE. Also, as previously describe in clade C isolates, we found no C/EBP binding site directly upstream of the enhancer region in CRF22_01A1 specimens. In our study, one-third of CRF02_AG LTRs displayed three NF-kB sites which have been mainly described in clade C isolates. Overall, the number, location and binding patterns of potential regulatory elements found along the U3R might be specific to some HIV-1 strains such as clade F2, CRF02_AG, CRF01_AE and CRF22_01A1. These features may be worth consideration as they may be involved in distinctive regulation of HIV-1 transcription and replication by different and diverse infecting strains.

## Introduction

The high diversity of HIV-1 has led to its classification into four groups named M, N, O and P. Group M which is the most prevalent worldwide has been further subdivided into at least nine genetically distinct subtypes or clades (A-D, F-H, J, and K), several circulating recombinant forms (CRFs) and unique recombinant forms (URFs) [1]. Several studies have reported the impact of HIV-1 strains on transmission, replication, pathogenesis, diagnosis and response to therapy [2, 3]. Thus, subtype A was shown to be less pathogenic than non-A subtypes and to have a lower replication rate than subtype C [4, 5]. The heterosexual transmission rate of HIV-1 appeared to be higher in subtype A compared with D, and in CRF01_AE compared with subtype B [6, 7]. Subtype D has been associated with faster disease progression compared with subtype A, C and CRFs [8–10]. The overall influence of inter- and intra-clade genetic variability on the biological properties of HIV-1 is not fully understood. However, there is increasing evidence that the genetic diversity of the LTR has the potential to affect the replication rate and expression of HIV-1 [11–14]. For example, it was shown that a single mutation changing one of the two canonical targets for NF-κB into a binding site for GABP transcription factor, results in higher replication rate and transmission efficiency of CRF01_AE compared with subtype B [15]. Also, mutations in the sequence motifs targeted by the CCAAT/enhancer-binding proteins (C/EBP) and specificity proteins 1 (Sp1) were correlated with disease severity and neurologic impairment [16, 17].

During the life cycle of HIV-1, transcriptional regulation of the integrated provirus is a key step ahead of viral replication and expression. This early step is regulated by a synergistic interaction of host transcription factors and viral proteins that bind to specific targets in the 5’LTR of the HIV-1 genome. The HIV-1 5’LTR is a complex structure of approximately 640 bp in length with a high concentration of transcription factor binding sites (TFBS). It has been divided into the U3, R and U5 functional regions. The U3 region located upstream of the transcription start site was subdivided into the modulatory (nt -454 to -104), the enhancer (nt -105 to -79) and the promoter (nt -78 to -1) segments [13, 18]. The modulatory segment contains numerous TFBS [19], and in certain HIV-1 variants, this segment is extended with an insert of 15 to 34 nucleotides known as the Most Frequent Naturally occurring Length Polymorphism (MFNLP) [20–22]. The enhancer region contains binding sites for the NF-kB family of transcription factors, and the promoter region carries the TATA box and three binding sites for Sp1 [18]. Following the promoter, the R region which is, by definition, the transcription initiation site comprises the trans-activating responsive (TAR) element that mediates activation of transcription through its binding to the viral Tat protein [23, 24], and the polyadenylation signal (poly A) involved in the addition of the 3'-poly (A) tail [25]. Along with these elements, various binding sites for different families of transcription factors have also been described in the R region [26–29]. Interaction between the TAR element and the viral protein Tat is required to enhance transcriptional elongation and gene expression of HIV-1 [30, 31]. However, prior to the presence of Tat, the 5’LTR is able to support basal transcription that results in the production of short transcripts allowing viral proteins such as Tat to be made [31]. This capacity of the 5’LTR to control HIV-1 basal transcription is driven by TFBS in the U3 promoter and enhancer segments primarily [31], but also involves cooperative action of regulatory elements in the U3 modulatory segment [32]. Besides its role in transcription, the U3 and the R region of the 5’LTR (U3R) have also been involved in HIV-1 replication, expression and silencing [33–38]. Therefore, characterization of the U3R from different HIV-1 strains is of great importance as it might contribute to a better understanding of the influence of HIV-1 variants on the fitness, pathogenicity and disease progression. To date, only few studies have investigated the genetic diversity the HIV-1 5’LTR in non-B and non-C strains and in most of them the common approach was the clone-based Sanger sequencing methodology [39–41]. This method is costly, time-consuming and labor intensive. Also, it may be limited by preferential selection that can occur during molecular cloning and only a few hundred clones can be generated and sequenced [42, 43]. Next-generation sequencing (NGS) technology is a relevant alternative to overcome these limitations by allowing massively parallel amplification at high coverage of thousands of reads per base pair. Thus, NGS provides the potential to reduce the time and facilitate the detection of intra-specimen variability without the need for cloning PCR amplicons before sequencing. The main objective of this study is to describe the genetic variability of the U3R from viruses representing various clades and diverse CRFs isolated from plasmas samples, by using NGS Illumina sequencing technology.

## Materials and methods

### Specimens and RNA isolation

Sixty five high titer virus derived from de-identified plasma specimens obtained from DUKE EQAPOL viral diversity program [44], and five CRF02_AG virus isolated from stored and de-identified plasma specimens [45–47] as previously described [48] were selected for this study. The study was approved by the Duke University Institutional Review Board for clinical investigation (Pro0029507) and the Review Board of the US HHS/Food and Drug Administration Research in Human Subjects Committee (exempt reference number 01-044B). The selected seventy virus stocks included clade B (n = 18), clade A1 (n = 10), clade C (n = 9), clade D (n = 8), clade F2 (n = 3), clade G (n = 4), CRF02_AG (n = 9), CRF01_AE (n = 5), CRF22_01A1 (n = 4). Viral RNA was extracted using Virus QIAamp Viral RNA Mini Kit (QIAGEN) according to the manufacturers’ instructions.

### Primer design

References and consensus sequences representing the first 1000 nucleotides of the HIV-1 genome from multiple subtypes and CRFs were downloaded from Los Alamos National Laboratory HIV database and aligned in MEGA version 7 software [49]. Regions with the highest degree of conservation were screened and used to design PCR primers that were optimized for melting temperatures, GC content and reduced hairpin and/or primer dimer formation (NCBI/primer3-BLAST; SMS). Due to mismatches in some strains, selected primers were designed with degenerate nucleotides to match all group M isolates.

### cDNA synthesis, PCR amplification and purification

Reverse transcription was performed by using Superscript III First-strand synthesis System for RT-PCR (Invitrogen 18080–051). The cDNA was amplified by touchdown (TD) polymerase chain reaction (PCR) procedure with 2 rounds of amplification. Each amplification run was performed in a 50μl reaction volume using Promega PCR master mix (Promega, Madison, WI, USA cat M7505) supplemented with 0.4μM of primer Table 1. Cycling conditions for both outer and nested TD-PCR was as follows: initial incubation at 95°C for 3 min followed by 10 cycles at 95°C for 30 s, 64°C for 30 s with a 1°C decrement per cycle, and 72°C for 50 s. Subsequently, 25 cycles were performed at 95°C for 30 s, 55°C for 30 s, and 72°C for 50 s followed by a final extension of 5 min at 72°C. The second round PCR amplicons were separated by gel electrophoresis. The expected PCR product size of about 850 pb were excised from the gel and purified using QIAquick Gel Extraction Kit (Qiagen).

**Table 1:** Primers used for amplification of the 5’end of the HIV-1 genome.

Primers	Reaction	Sequence (5’to 3’)	Position ^a^
HF01F (+)	cDNA	TGGAAGGGCTAATTTGGTCCCA	1–22
UNINEF 7’ (-)		GCACTCAAGGCAAGCTTTATTGAGGCTT	9605–9632
HF01Fd (+)	1^st^ PCR	TGGAWGGGYTAATTTGGTCCCA	1–22
RVN910_M (-)		GCTCCCTGCTTGCCCATACT	891–910
5'NCF1_M (+)	2^nd^ PCR	GGMTWCTTCCCTGATTGGCA	66–85
PBSBR (-)		CTTAATACCGACGCTCTCGCACCCAT	790–815

(+), forward primer; (-) Reverse primer;

^a^ Position according to the HXB2 coordinates

### Ultra-deep sequencing

Miseq (Illumina, San Diego, CA) sequencing of the gel purified PCR product was carried out at the core facility of the Food and Drug Administration (FDA, White oak, MD, USA). Briefly, concentration of purified amplicons was measured by using Qubit dsDNA BR Assay System (Covaris, Woburn, MA, USA) and (2 ng of DNA purified product was processed for NGS library preparation using the Nextera XT DNA Sample Preparation Kit. Specimens were run in the Miseq instrument using a MiSeq v2 kit (500 cycles) to produce paired-end reads of approximately 250pb. After automated cluster generation in MiSeq, the sequencing was processed and fastq files were generated.

### Sequence analysis

All fastq files were imported into CLC Genomics Workbench version 9.0 (CLC bio/Qiagen, Aarhus, Denmark) using default option. Reads were paired and trimmed to obtain reads with an average Phred score ≥ 20. Contigs were generated using de novo assembly tools with the default options. The reads were mapped back to contigs using previous published parameters [50]. For characterization of the 5’LTR region, the minimum contig length was set at 400bp. For each specimen, contigs with a minimum coverage of 1000 reads were exported from the assembly and used for further analysis. The subtype of each full length LTR contig was assigned using COMET HIV-1 subtype tool [51]. Sequences from the same subtypes were aligned in MEGA 7 using the 5’end of HXB2 sequence (from nucleotide 76 to 850, K03455) as a reference to position the U3R sequence and to locate regulatory motifs. Mutations were identified by comparing sequences from each HIV-1 studied strain to the reference HXB2 and within matched subtype. Identification of potential TFBS was based on similarity with a reported binding site pattern and prediction with the Match 1.0 Public program available online (http://gene-regulation.com/pub/programs.html). A pairwise comparison in CLC Genomics Workbench was used to calculate the percentage of identity.

## Results

### Subtype assignment

Near full length LTR sequences were not obtained for 11% (8/70) of selected specimens due to failure in amplification and /or sequencing. From the 62 specimens successfully sequenced, more than one contig was obtained for several specimens. Thus, we retrieved a total of 80 contigs that were subtyped as follows: clade B (n = 20), CRF12_BF (n = 5), clade C (n = 8), clade D (n = 10), clade F2 (n = 5), clade G (n = 3), clade A1 (n = 10), CRF02_AG (n = 9) and CRF01_AE (n = 10). For majority of the sequences analyzed, the subtype assigned to the 5’LTR of the HIV-1 isolate was concordant with its previously reported primary genotype [44–46, 52]. However, we found that additional contigs from 5 clade B specimens were subtyped as CRF12_BF (BF), one of the two contigs from a CRF02_AG (AG) specimen was assigned to be F2, and all the LTR sequences retrieved from the CRF22_01A1 (01A1) specimens were subtyped as CRF01_AE (AE) Table 2.

**Table 2:** Specimens description and LTR subtype assignment.

Original ID	Country of origin	Primary genotype	Source Genbank	LTR ID	LTR subtype	Genbank
DEMB10US007	USA	B	KC473828	DEMB10US007	B	MH045863
DEMB10US011	USA	B	KC473830	DEMB10US011	B	MH045864
DEMB10US003	USA	B	KC473826	DEMB10US003	B	MH045865
DEMB10US004	USA	B	KC473827	DEMB10US004	B	MH045866
DEMB11US004	USA	B	KC473832	DEMB11US004	B	MH045867
DEMB10US009*	USA	B	KC473829	DEMB10US009_1	B	MH045868
				DEMB10US009_2	CRF12_BF	MH045869
DEMB05FR001	France	B	JX140652	DEMB05FR001	B	MH045870
DEMB08FR002*	France	B	JX140654	DEMB08FR002_1	B	MH045871
				DEMB08FR002_2	B	MH045872
DEMB10CN002*	China	B	JX140658	DEMB10CN002_1	B	MH045873
				DEMB10CN002_2	B	MH045874
DEMB10VE001	Venezuela	B	JX140659	DEMB10VE001	B	MH045875
DEMB03JP004*	Japan	B	KC473846	DEMB03JP004_1	B	MH045876
				DEMB03JP004_2	B	MH045877
				DEMB03JP004_3	CRF12_BF	MH045878
DEMB10ES003*	Spain	B	KC473843	DEMB10ES003_1	B	MH045879
				DEMB10ES003_2	CRF12_BF	MH045880
DEMB10ES002	Spain	B	KC473842	DEMB10ES002	B	MH045881
DEMB09ES007	Spain	B	KC473841	DEMB09ES007	B	MH045882
DEMB12JP001	Japan	B	KF716498	DEMB12JP001	B	MH045883
DEMB09FR001*	France	B	KF716494	DEMB09FR001_1	B	MH045884
				DEMB09FR001_2	CRF12_BF	MH045885
DEMB09BO001*	Bolivia	B	JX140656	DEMB09BO001_1	B	MH045886
				DEMB09BO001_2	CRF12_BF	MH045887
DEMC08ZA011	South Africa	C	JX140666	DEMC08ZA011	C	MH045888
DEMC09MW007	Malawi	C	KP109524	DEMC09MW007	C	MH045889
DEMC09MW009	Malawi	C	KP109526	DEMC09MW009	C	MH045890
DEMC00IN009	India	C	KP109484	DEMC00IN009	C	MH045891
DEMC12ZA087	South Africa	C	KP109516	DEMC12ZA087	C	MH045892
DEMC00IN006*	India	C	KP109481	DEMC00IN006_1	C	MH045893
				DEMC00IN006_2	C	MH045894
DEMC00IN007	India	C	KP109482	DEMC00IN007	C	MH045895
DEMD07UG007	Uganda	D	KF716503	DEMD07UG007	D	MH045896
DEMD07UG002*	Uganda	D	KC596071	DEMD07UG002_1	D	MH045897
				DEMD07UG002_2	D	MH045898
DEMD08UG001	Uganda	D	KC596072	DEMD08UG001	D	MH045899
DEMD10UG004*	Uganda	D	KF716479	DEMD10UG004_1	D	MH045900
				DEMD10UG004_2	D	MH045901
DEMD11UG003*	Uganda	D	KF716480	DEMD11UG003_1	D	MH045902
				DEMD11UG003_2	D	MH045903
				DEMD11UG003_3	D	MH045904
DEMD10CM009	Cameroon	D	JX140670	DEMD10CM009	D	MH045905
DEMF110ES001	Spain	F1	JX140671	DEMF110ES001	F2	MH045906
DEMF210CM001*	Cameroon	F2	JX140672	DEMF210CM001_1	F2	MH045907
				DEMF210CM001_2	F2	MH045908
DEMF210CM007	Cameroon	F2	JX140673	DEMF210CM007	F2	MH045909
NYU6542*	Cameroon	CRF02_AG	NA	NYU6542_1	F2	MH045910
				NYU6542_2	CRF02_AG	MH045911
DE00208CM004	Cameroon	CRF02_AG	JX140647	DE00208CM004	CRF02_AG	MH045912
DE00208CM001	Cameroon	CRF02_AG	JX140646	DE00208CM001	CRF02_AG	MH045913
MDC-021	Cameroon	CRF02_AG	NA	MDC-021	CRF02_AG	MH045914
MDC-024	Cameroon	CRF02_AG	NA	MDC-024	CRF02_AG	MH045915
MDC-046	Cameroon	CRF02_AG	NA	MDC-046	CRF02_AG	MH045916
MDC-055*	Cameroon	CRF02_AG	NA	MDC-055_1	CRF02_AG	MH045917
				MDC-055_2		MH045918
MDC-067	Cameroon	CRF02_AG	NA	MDC-067	CRF02_AG	MH045919
DEMG09KE001	Kenya	G	KF716477	DEMG09KE001	G	MH045920
DEMG09ES002	Spain	G	JX140675	DEMG09ES002	G	MH045921
DEMG10CM008	Cameroon	G	JX140676	DEMG10CM008	G	MH045922
DE00109CN003	China	CRF01_AE	KC596061	DE00109CN003	CRF01_AE	MH045923
DE00109CN004	China	CRF01_AE	KC596062	DE00109CN004	CRF01_AE	MH045924
DE00110CN001	China	CRF01_AE	KC596063	DE00110CN001	CRF01_AE	MH045925
DE00111CN003	China	CRF01_AE	KC596065	DE00111CN003	CRF01_AE	MH045926
DE00111CN002	China	CRF01_AE	KC596064	DE00111CN002	CRF01_AE	MH045927
DE02210CM010	Cameroon	CRF22_01A1	KF716460	DE02210CM010	CRF01_AE	MH045928
DE02210CM011	Cameroon	CRF22_01A1	KF716461	DE02210CM011	CRF01_AE	MH045929
DE02210CM014	Cameroon	CRF22_01A1	KF716463	DE02210CM014	CRF01_AE	MH045930
DE02210CM012*	Cameroon	CRF22_01A1	KF716462	DE02210CM012_1	CRF01_AE	MH045931
				DE02210CM012_2		MH045932
DEMA03RW001	Rwanda	A1	KF716499	DEMA03RW001	A1	MH045933
DEMA11KE001	Kenya	A1	KF716475	DEMA11KE001	A1	MH045934
DEMA110UG001	Uganda	A1	KF859745	DEMA110UG001	A1	MH045935
DEMA108RU003	Russia	A1	KF716491	DEMA108RU003	A1	MH045936
DEMA108RU004	Russia	A1	KF716492	DEMA108RU004	A1	MH045937
DEMA106ES002	Spain	A1	JX140651	DEMA106ES002	A1	MH045938
DEMA110UG009	Uganda	A1	KF716486	DEMA110UG009	A1	MH045939
DEMA105TZ001	Tanzania	A1	JX140650	DEMA105TZ001	A1	MH045940
DEMA07RW002*	Rwanda	A1	KP109528	DEMA07RW002_1	A1	MH045941
				DEMA07RW002_2		MH045942

NA, not available;

*highlighted in gray are specimens in which more than one contig was retrieved

### Variability of the U3R region of the 5’LTR

Compared to HXB2, the U3 region was the most conserved in clade B with about 88% nucleotide identity versus less than 80% in average for all the other strains. The variability of the U3 region was less than 12% between sequences from the same strains and increased to an average of 23% between sequences from different strains. For most of our studied strains, the highest variability was found in the U3 modulatory segment with less than 75% of nucleotide identity. Nevertheless, the enhancer segment of clade C 5’LTRs share less than 65% of identity with the other strains. Although the number of mutations were higher in the modulatory segment than in the other segments of the U3 region, it also appears to be well conserved within sequences from the same HIV-1 strain (>85% of nucleotides identity). In the enhancer segment the intra-strain diversity varied from 2% (CRF01_AE) to 26% (CRF02_AG), while it was about 6% in the promoter segment. With an average nucleotide identity of 85%, this latter segment of the U3 region displayed few changes when sequences from different strains were compared.

### Variability of the U3 region

#### U3 modulatory spanning nucleotides -378 to -176

**Fig 1:**
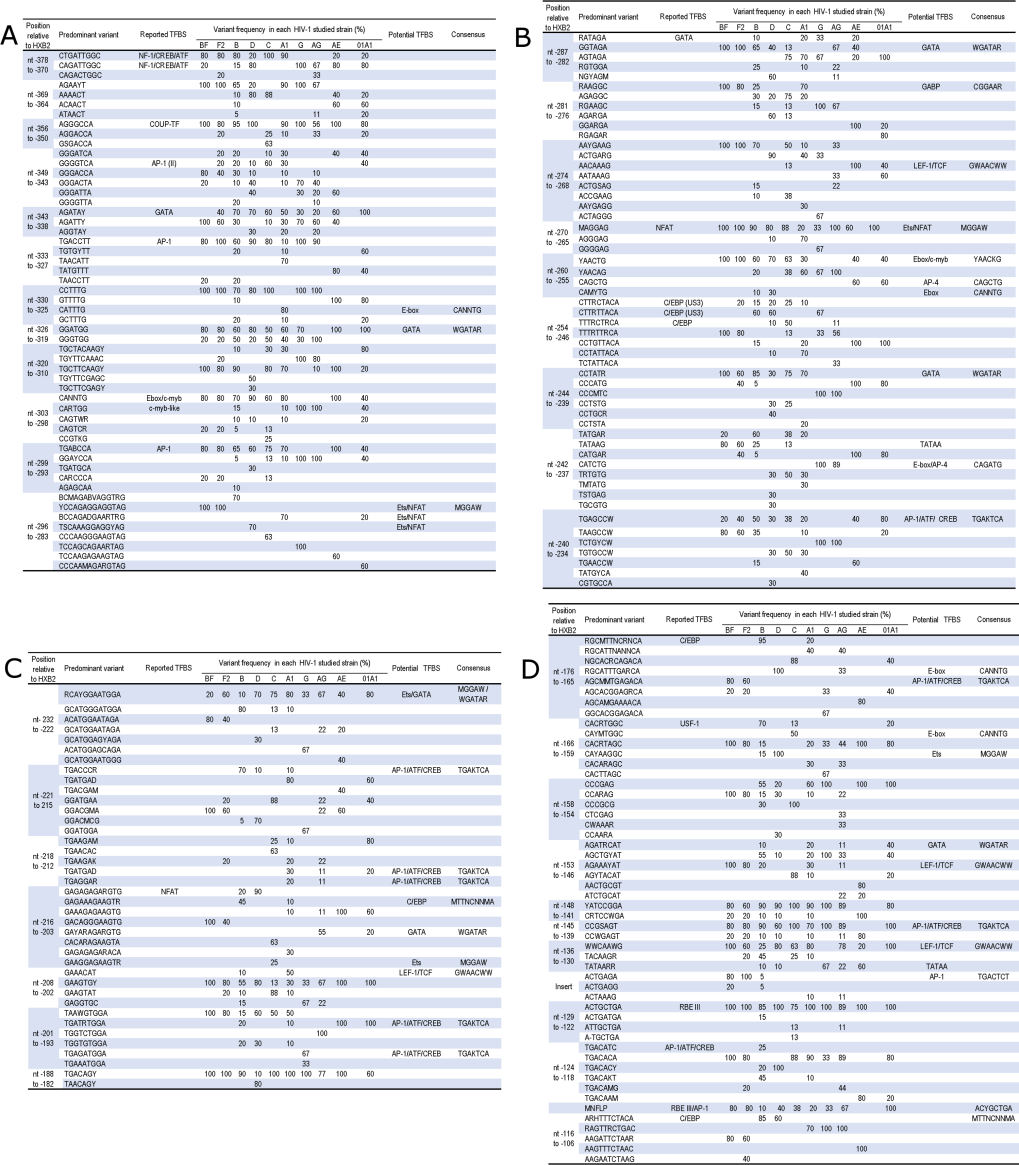
Variability in the U3 modulatory region of diverse HIV-1 subtypes and CRFs. N = any base; W = A or T; R = A or G; Y = C or T; K = G or T; M = A or C; S = G or C; D = A, G or T; H = A, C or T; B = C, G or T.

Numerous transcription factors have been described in this portion of the U3 modulatory segment [13]. Nucleotide sequence from -378 to -364 which encompasses targets for proteins related to the NF-1 and CREB/ATF family of transcription factors [53, 54], include the CTGATTGGC motif (nt -378 to -370) that was conserved in 56% of the sequences analyzed. Its variant CAGATTGGC was mainly observed in the CRFs as well as in clades G and D specimens, while CAGACTGGC was shown in 1/3 of the CRF02_AG specimens (Fig 1A). The adjacent sequence (nt -369 to -364) with the consensus motif AGAA(C/T)T was conserved in 55% of the sequences analyzed, while other variants such as ACAACT were mainly found in CRF01_AE and CRF22_01A1 viruses (Fig 1A). TFBS such as COUP-TF, AP-1, ETS-1 and GATA have been reported within the sequence spanning nt -356 to -325 [55–57]. In our dataset, the first half of the binding site for COUP/AP-1 (AGGGCCA; nt -356 to -350) was highly conserved (80%) with few variants such as the consensus G(G/C)GACCA found in clade C LTRs (Fig 1A). On the contrary, the second half of this TFBS (nt -349 to -343) which includes a functional target for AP-1 family of proteins (AP-1 (II)), was more variable (Fig 1A). AP1 (II) overlaps a GATA binding site (nt -343 to -338) with the motifs AGATT(T/C) mostly found in F2, CRF12_BF, G and CRF02_AG LTRs, and AGATA(T/C) predominant in all the other studied strains. Further downstream (nt -333 to -327), the TGACCTT consensus for the reported AP-1(I), was found in all the strains studied except in CRF01_AE, CRF22_01A 1 and in clade A1 viruses. Indeed, the AP-1 (I) variant TATGTTT was shared by 80% of CRF01_AE and 40% of CRF22_01A1 specimens. In the remaining sequence from CRF22_01A1 viruses, the consensus sequence TGTG(T/C)TT was found. In clade A1 LTRs, the variant TAACATT generates with its 3’end overlapping nucleotides a CATTTG motif (nt -330 to -325), that matches the E-box consensus CANNTG (Fig 1A).

Contiguously, the GATA-like binding site GGATGG (nt -326 to -319) [58], was mutated to GGGTGG in CRF02_AG LTRs and in some sequences from various other strains (Fig 1A). The consensus TGCTACAAG(C/T) (nt -320 to -310) of the first half of the binding site for the negative regulator of transcription (NRT-1)[59], was mainly conserved in CRF22_01A1 (80%) isolates. It was changed to a TGCTTCAAG(C/T) consensus in a majority of non -CRF02_AG, non- clade G and D specimens, and to TG(C/T)TTCAAAC variant in clade G and CRF02_AG LTRs. The second half of the NRT-1 was reported to include an E-box/c-Myb and AP-1 sites [60]. In our study, the E-box consensus (CANNTG, nt -303 to -298) was not found in clade G and CRF02_AG LTRs. It was replaced by the non-specific variant CA(A/G)TGG also shared with others strains such as CRF22_01A1 isolates (Fig 1A). This latter variant also modified the overlapping AP-1 motif (nt -299 to -293). The contiguous GATA target [60, 61], displayed two major variants in our study (Fig 1A), GGTAGA (45%) and AGTAGA (26%) that were closely related but not identical to the canonical (A/T)GATA(A/G) binding site for GATA factors. Nucleotides spanning the AP1-1 and GATA binding sites (nt -296 to -283) lead to a consensus with a fairly high variability in LTRs from clades B and A as well as CRF02_AG specimens, but with some specific patterns in the other strains analyzed. The HXB2 sequence GCCAGAGAAGTTAG was mutated to CCCAAGGGAAGTAG in clade C, to T(G/C)CAAAGGAGG(C/T)AG in clade D, and to TCCAAGAGAAGTAG in CRF22_01A1 isolates. These variants probably contain specific TFBS, as observed with the clade D included motif AAGGAG that matches a recognition site for Ets-2 [62].

In the subsequent sequence, motifs such as (G/A)AAGGC (nt -283 to -277), fitting the canonical motif for GABP factors [63], was observed in all F2 and CRF12_BF as well as in A1 (70%) and B (25%). The AACAAAG motif (nt -274 to -268) conformed to the LEF-1/TCF consensus [64], was conserved only in LTRs from CRF01_AE (100%) and CRF22_01A1 (40%). In 60% of CRF22_01A1 and in 33% of CRF02_AG isolates, the LEF-1/TCF motif was changed to a homologue of the polyadenylation signal motif AATAAA (Fig 1B). The overlapping consensus (A/C)AGGAG (nt -270 to -265) which resembles a binding site for NFAT and Ets-2 factors [62, 65–67], was fairly well conserved across almost all our analyzed sequences except for majority of sequences from clades A1 and G isolates (Fig 1B). Overlapping the NFAT footprint [68], the consensus motif CAACTG (nt -260 to -255) perfect fit to the E-box /c-Myb consensus [69, 70], was mainly found in non-A1, non-G and non-CRFs specimens. In 60% of the sequence assigned to be CRF01_AE, we found the variant CAGCTG similar to the recognition site for the AP4 factor [71]. On the other hand, the c-Myb variant (C/T)AACAG, predominant in sequences from CRF02_AG (100%), G (67%) and A1 (60%) specimens, was also found in clades C (38%) and B (20%).

Although it displayed one nucleotide mismatch with the (C/A)TTNCNN(C/A)A consensus described as the best fit for the binding of the C/EBP family of transcription factors [72–74], the sequence CTTGTTACA in HXB2 (nt -254 to -246) was reported as a C/EBPβ target (US3). In our study, the US3 consensus (CTTNTNNCA) was found in at least 60% of clades B, D and G assigned LTRs, and the variant TTTNCNNCA mainly found in clade C LTR was also shown to be able to bind C/EBP proteins [74–76]. In most of the other studied strains, variants of the US3 generally included more than one nucleotide mismatches with the C/EBP consensus motif (Fig 1C). The adjoining sequence (nt -244 to -234) seemed to include various overlapping TFBS with some strain specific motifs (Fig 1C). Thus, at the relative location of the GATA-like motif CCTAT(G/A) (nt -244 to -239) that was found in non-clade G and non–CRFs isolates, the CCCATG variant was shown in LTRs subtyped as CRF01_AE only, while CCCATC was found in clade G and CRF02_AG only. Subsequently, we observed a CREB/ATF/AP-1-like binding site in several studied isolates, except for clade G and CRF02_AG. In these latter specimens, we found an E-box with the sequence motif CATCTG. Majority of sequences subtyped as F2 (60%), CRF12_BF (80%) as well as some clade B (25%) displayed a TATAA motif within nucleotides spanning this location (Fig 1C). Further downstream, the nucleotide sequence includes a potential Ets/GATA- like element (nt- 232 to -222) and other various potential TFBS such as TGACCC(A/G) in clade B or TGA(G/T)GA(A/G) in clade A1 which resemble were a CREB/ATF/AP-1 target (nt -221 to -212).

The sequence GAGAGAGAAGTG in HXB2 (nt -216 to -203), reported to encompass a second NFAT footprint in this segment of the U3 region [65, 68], was mainly conserved in clade D isolates. In our dataset, this NFAT footprint displayed strain specific mutations generating motif that might potentially be recognized by other transcription factor (Fig 1C). For example, the GAGAAAGAAGT(G/A) consensus was mainly found in clade B LTRs (45%) include C/EBP-like motif, while the GA(C/T)A(G/A)A(G/A)ARGTG variant predominant in CRF02_AG isolates includes a GATA-like motif. The highest variability of this NFAT footprint was found in clade A1 isolates in which we noticed that 50% of the variants contain a GAAACAT motif (nt -208 to -202) closely related to the LEF-1/TCF recognition site [64]. The adjacent motif TAGAGTGGA (nt -201 to -193) displayed some specific motifs in most of the HIV-1 non-B strains with some the variant containing an AP-1/ATF/CREB-like motif (Fig 1C). The following nucleotide sequence (nt -188 to -182) was characterized by low intra-clade variability and majority of the variants found contain a motif homologous to the AP-1/ATF/CREB family of proteins target (Fig 1C).

#### U3 modulatory spanning nucleotides -176 to -106

Several consensus binding sites for transcription factors such as C /EBP, USF-1, Ets, LEF-1 and RBF-2 have been described within the nucleotide sequence from position -176 to the end of the modulatory segment [41, 73, 77–79]. In this part of the U3 region, the US2 and US1 targets for C/EBP proteins were reported within the HXB2 sequence AGCATTTCATCA (nt -176 to -165) and AGCTTGCTACA (nt -116 to -106) respectively [80]. In our study, the consensus motif (A/G)GC(A/C)TTNC(A/G)NCA found in clades B (95%) and A1 (20%) was the only US2 variant conformed to the canonical binding site for C/EBP proteins. Non-canonical distinct variants were observed among non-B strains and some of them displayed up to three nucleotides mismatches with the consensus (Fig 1D). The consensus (A/G)GCATTNANNCA was found in about 40% of A1 and 45% of CRF02_AG LTRs, while NGCAC(A/G)CAGACA predominant in sequences from clade C (88%) was also found in LTRs from CRF22_01A1 specimens (40%). In our study, the sequence CGCAGACA included in this latter US2 variant was no longer strictly associated to clade C isolates as previously reported [39, 81], as it was also found in the emerging CRF22_01A1 recombinant. On the other hand, we observed that the US2 consensus AGCA(C/A)GAAAACA was only displayed by CRF01_AE specimens. In all the clade D and in some CRF02_AG LTRs, the US2 variant (A/G)GCATTTGA(A/G)CA includes the E-box motif CATTTG similar to what we reported earlier in clade A1 viruses (nt- 330 to -325); whereas the AGC(C/A)(C/A)TGAGACA consensus in CRF12_BF (80%) and F2 (60%) LTRs, contains a TGAGACA motif closely related to an AP-1/CREB/ATF target.

Overlapping with the US2 target, the nucleotide sequence CACATGGC in HXB_2_ (nt -166 to -159) contains the well described E-box consensus motif CAC(G/A)TG recognized by bHLHzip proteins such as USF-1 or TF3 [75, 82, 83]. This USF-1 consensus was mainly found in clade B LTRs (70%), in some CRF22_01A1 (20%), and in few clade C isolates (13%). At this position, additional E-boxes with non-USF-1 consensus motifs were found in clade C (50%), while none of the variants found in the other studied strains match the CANNTG consensus (Fig 1D). According to a previous study, these non-CANNTG variant do not interact with a recombinant USF-1 factor in vitro [84]. We noticed that the A5T mutation of the USF-1 variant CACAAGGC displayed by the clade D and few clade B LTRs, generates an Ets-like core motif (AAGGC). The sequence downstream of the USF-1 motif (nt -153 to -146), includes a potential GATA motif with a high variability across our dataset (Fig 1D). In this sequence, we also observed that the consensus AGAAA(C/T)AT predominant in F2 and CRF12_BF LTRs (Fig 1D), contains a potential recognition site for TCF/LEF family of proteins [64].

Contiguously, the consensus motif (C/T)ATCCGGA (nt -148 to -141) binding site for RBF-1/Ets-1 was found to be very well conserved across all our studied HIV-1 strains except for CRF01_AE isolates mainly in which it was changed to a C(G/A)TCC(T/A)GA variant as previously described [14, 40, 41]. Here, the RBF-1/Ets-1 target overlaps two major consensus motifs CCG(G/C)AGT and CC(T/A)GAGT (mainly CRF01_AE) in 79% and 14% of the analyzed LTRs respectively (Fig 1D). These two consensuses contain a motif homologous to the recognition site the ATF/CREB/AP-1 family of transcription factors. The canonical consensus for LEF-1/TCF factors commonly reported in proximity of the Ets-1 target [82], was found in about 60% of the non-B, non-G and non-CRF01_AE specimens in our study. For this TFBS, a TATAA(A/G)(A/G) variant which includes a TATAA motif was predominant in G (67%) and CRF01_AE (60%) isolates. Nonetheless, this TATAA motif has been shown to be non-functional in CRF01_AE isolates [41]. The highest variability was observed in clade B LTRs where only 25% of the TCF/LEF variants matched the canonical motif. Adjacent to the binding site for TCF/LEF, an insert of 7 nucleotides with the consensus motif ACTGAGA was specifically found in F2 and CRF 12_BF sequence. In this study, we noticed that this insert reported as a partial RBE site motif [41], was closely related to the AP-1- like element involved in the expression of the tumor suppressor protein p53 [85].

Further downstream, the ACTGCTGA motif of the RBEIII site (nt -129 to -122) targeted by the RBF-2 complex [22, 77], was particularly well conserved (93%) across HIV-1 clades and CRFs in our study. This RBE III intersects an AP1-like element (nt -124 to -118) that was conserved in only 25% of the clade B LTRs. Besides the most frequent variant TGACACA predominant in sequences from non-B, D, G and non-CRF01_AE isolates, the AP-1-like sequence displayed some strains distinctive motifs (Fig 1D). It was followed by the MFNLP which in accordance with previous reports [21, 22], was displayed by about 33% of the LTRs in our study. Commonly observed in LTRs from CRF22_01A1 specimens as well as in LTRs subtyped as F2, CRF12_BF (80%) and CRF02_AG (67%), this insert was less frequent (≤ 40%) in clades A1, G, C and D. It was scarcely found in LTRs subtyped as B, and was totally absent in the LTRs from CRF01_AE isolates. The MFNLP includes a duplicate of the RBEIII and/or the AP-1-like motif along with a GATA-like motif in the LTRs subtyped as CRF02_AG, CRF12_BF and F2 or a C/EBP-like motif in LTRs from clade D isolates. At the end of the modulatory segment, the US1 consensus A(A/G)(C/A/T)TTTCTACA found in clades B (70%) and D (80%) LTRs, was the only in our dataset to be conformed to a reported binding site for C/EBP proteins. In all the other analyzed 5’LTR sequences, the following variants were found: (A/G)AGTT(G/A)CTGAC in A1, G, and CRF02_AG, AAGATTCTAA(G/A) in F2 and CRF12_BF, while AAGTTTCTAAC which overlaps a TCF/LEF-like motif (CTAACTA) was found in CRF01_AE specimens (Fig 1D). In our study, there was no US1 variant in sequences from clade C isolates as previously reported [76], neither in LTRs from CRF22_01A1 specimens.

#### U3 enhancer spanning nucleotides -104 to –80

**Fig 2:**
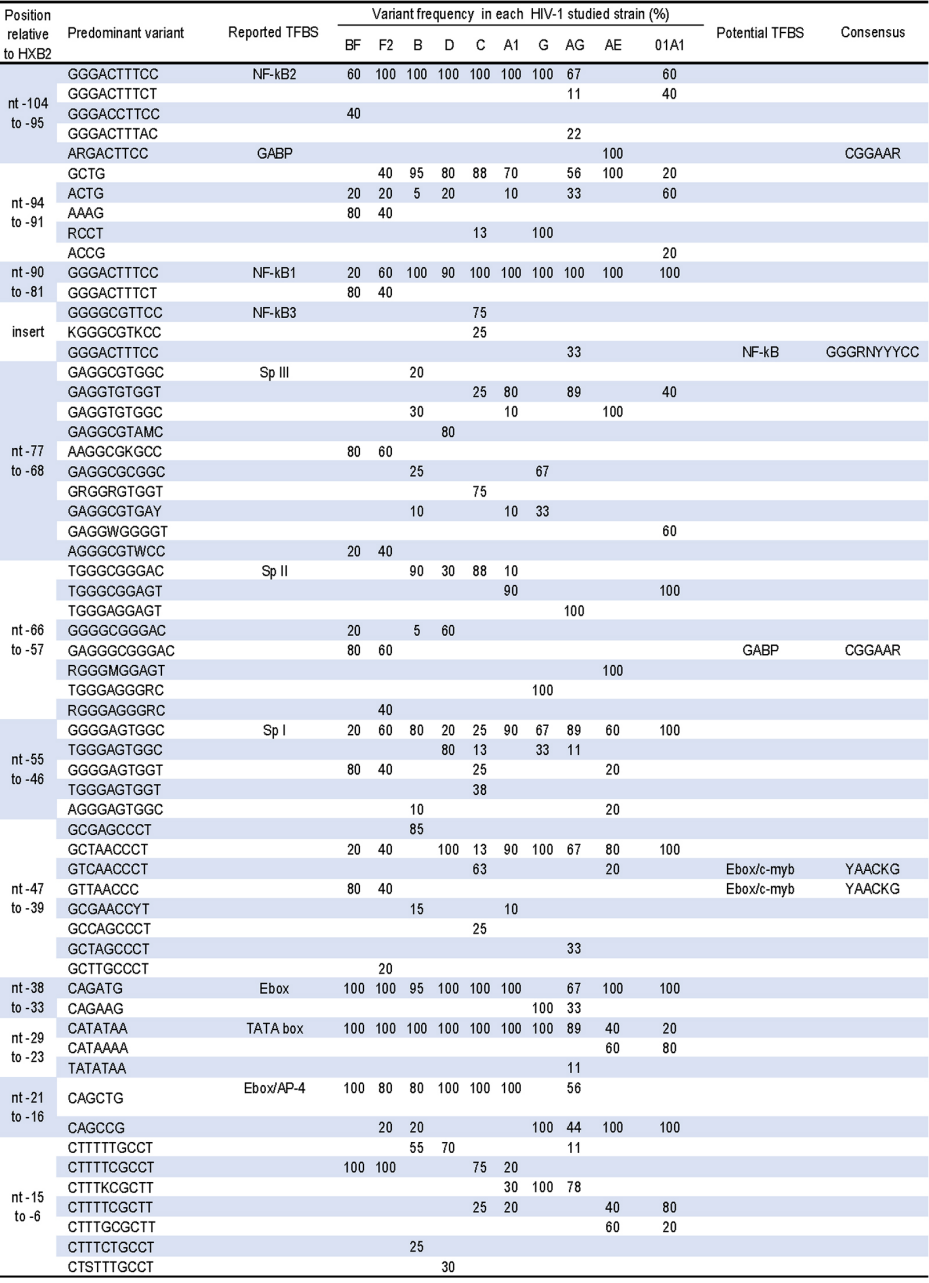
Variability in the U3 enhancer and promoter region of diverse HIV-1 subtypes and CRFs. N = any base; W = A or T; R = A or G; Y = C or T; K = G or T; M = A or C; S = G or C; D = A, G or T; H = A, C or T; B = C, G or T.

The two canonical sequences GGGACTTTCC for the binding of NF-κB factor in the enhancer segment of the U3 region were very well conserved. Nevertheless, with an overall percentage of similarity of 85%, NF-κB2 (nt -104 to -95) was slightly less conserved than NF-κB1 (nt -90 to -81) which showed a nucleotide similarity of about 91% with HXB2. Besides the consensus A(G/A)GACTTCC specifically found in CRF01_AE specimens, we observed within the NF-κB2 site some point mutations such as C9A in 20% of CRF02_AG, T6C in 40% of CRF12_BF LTRs and C10T in 40% of the LTRs from CRF22_01A1 isolates (Fig 2). The C10T mutation was also the most frequent NF-κB1variant and was observed only in CRF12_BF (80%) and F2 (40%) LTRs. This C10T variant found was shown to have no impact on the affinity and the functionality of the NF-κB factors [86]. In our study, an additional binding site for NF-kB with the consensus motif GGGGCGTTCC was found in majority of clade C specimens as previously reported [15, 41] and with the canonical NF-kB motif in one-third of CRF02_AG LTRs.

The NF-κB2/ NF-κB1 interspace motif GCTG (nt -94 to -91) was overall well conserved. This spacer is embedded within the AP-2 recognition sequence CCGCTGGGGA [87, 88], and overlaps motif targeted by GABP factors. Indeed, it has been shown that GABP binds with high affinity to the variant CTTCCG of overlapping the NF-κB2 site of CRF01_AE viruses only; and with the low affinity to the variant CTTTCCG present in most of the other studied strains [15]. In our dataset, we observed three major variants of the interspace motif (Fig 2): 1) ACTG scattered among several specimens and predominant in CRF22_01A1, 2) (G/A)CCT found in clade G, and 3) AAAG found in CRF12_BF and F2 LTRs only. This latter variant, by overlapping NF-κB2, led to a CTTTCCAAA motif conformed to the canonical consensus for the binding of C/EBP proteins ((C/A)TTNCNN(C/A)A). Furthermore, we noticed that the mutation of the AP-2 target in CRF02_AG appeared only in LTRs containing an extra NF-κB site.

#### U3 promoter spanning nucleotides -78 to –1

Three sites named Sp III, Sp II and Sp I for the binding of Sp1 and related factors have been described along with the TATA box in this segment of the U3 region. In our study, the Sp binding sites displayed a high degree of intra-clade conservation and some inter-clade variability. Of the three, the sequence of the Sp III proximal to NF-κB1 displayed the highest variability (Fig 2). Thus, the HXB_2_ Sp III consensus motif GAGGCGTGGC (nt -77 to -68) was found in 20% of clade B LTR only, while specific variants, such as GAGGCGTA(A/C)C was found in clade D isolates, and GAAGGCG(T/G)GCC which include a GABP–like motif (GAAGGC) was found in F2 (60%) and CRF12_BF (80%) LTRs (Fig 2). For this TFBS, the variant GAGGTGTGGT (5T10T) predominant in LTRs from clades A1 and CRF02_AG viruses (≥ 80%) was also found in sequences from CRF22_01A1 (50%) and clade C (22%), and GAGGTGTGGC (5T) seen in all the LTRs from CRF01_AE was shared with clade B LTRs as previously observed [89, 90]. Overall, variability of the Sp III in our study was concordant with previous studies reporting a high conservation of the central Gs (GGCG) and the frequent C5T/A mutation [90, 91]. The HXB_2_ Sp II motif TGGGCGGGAC (nt -66 to -57), mainly conserved in clades B and C sequences was mutated to TGGGCGGAGT in LTRs from clade A1 (90%) and CRF22_01A1 (100%) viruses. In our study, Sp II strain specific variants such as TGGGAGGG(G/A)C, TGGGAGGAGT, and (G/A)GGG(A/C)GGAGT were respectively observed in LTRs from clade G, CRF02_AG and CRF01_AE specimens (Fig 2). These three consensus were not conformed to the GC box consensus (GGGCGGPuPuPy) reported as recognition site for Sp1 family of proteins [92]. Sp I proximal to the TATA box (nt -55 to -46), was the most conserved of the three targets for Sp proteins, with the consensus motif (G/T)GGGAGTGG(C/T) found in more than 90% of the sequences analyzed.

Overlapping with the Sp I site, the subsequent nucleotide sequence GCGAGCCCT in HXB_2_ (nt -47 to -39) was conserved in clade B LTRs only (Fig 2). In our study, this sequence displayed several variations such as GTCAACCCT predominant in clade C, GTTAACCC seen only in F2 (40%) and CRF12_BF (80%) sequences, and the consensus GCTAACCCT observed in most of the other studied LTRs. We noted that these variants contained a potential E-box/c-Myb element. Further downstream, the TATA box motif (TATAA) flanked at its 3’ and 5’ end by an E-box motif, was extremely well conserved in all our strains studied except for CRF01_AE and CRF22_01A1 where it was changed to TAAAA in 60% and 80% LTR sequence respectively (Fig 2A). The CAGATG motif of the 5’E-box predominant in almost all the LTRs analyzed, was mutated to CAGAAG in clade G (100%) and in CRF02_AG (33%) viruses. However, we noted that in these two HIV-1 strains, a motif similar to the 5’E-box was observed in the sequence adjacent to the US3 target located in the modulatory segment (nt -242 to -237). The 3’E-box consensus sequence CAGCTG reported as a binding site for bHLH factors such as AP-4 [71], was mainly conserved in non-G and non-CRFs specimens where the CAGCCG variant was predominant (Fig 2). Thus, unlike what was previously reported [41, 74], our study suggests that the variability of the 3’E-box motif might not be restricted to clade G and CRF01_AE isolates. The sequence between the 3’E-box and the transcriptional start site was reported to contain a target for Oct-1/Oct-2 factor [93]. In this sequence, the CTTTT(T/C)GCCT consensus (nt -15 to -6) was the major variant in non-G, non-A1 and non CRFs isolates (Fig 2), whereas CTTCTCGC(C/T)T consensus was shared by clade A1 (30%), clade G (100%) and CRF02_AG (78%) LTRs, and CTTT(G/T)CGCTT was predominant in CRF01_AE and CRF22_01A1 specimens.

### Variability of the repeat (R) region

The TAR element (nt 1 to 59) fold into a hairpin structure composed of a bulge, a loop and a stem. In our study, we found that all the constitutive elements of the TAR structure were affected by singles and clustered sets of mutations across different HIV-1 strains. In general, the stem displayed the highest variability followed by the bulge (nt 23 to 25, with the TCT motif in HXB_2_) and the loop (nt 30 to 35, with a CTGGGA motif in HXB_2_).

**Fig 3:**
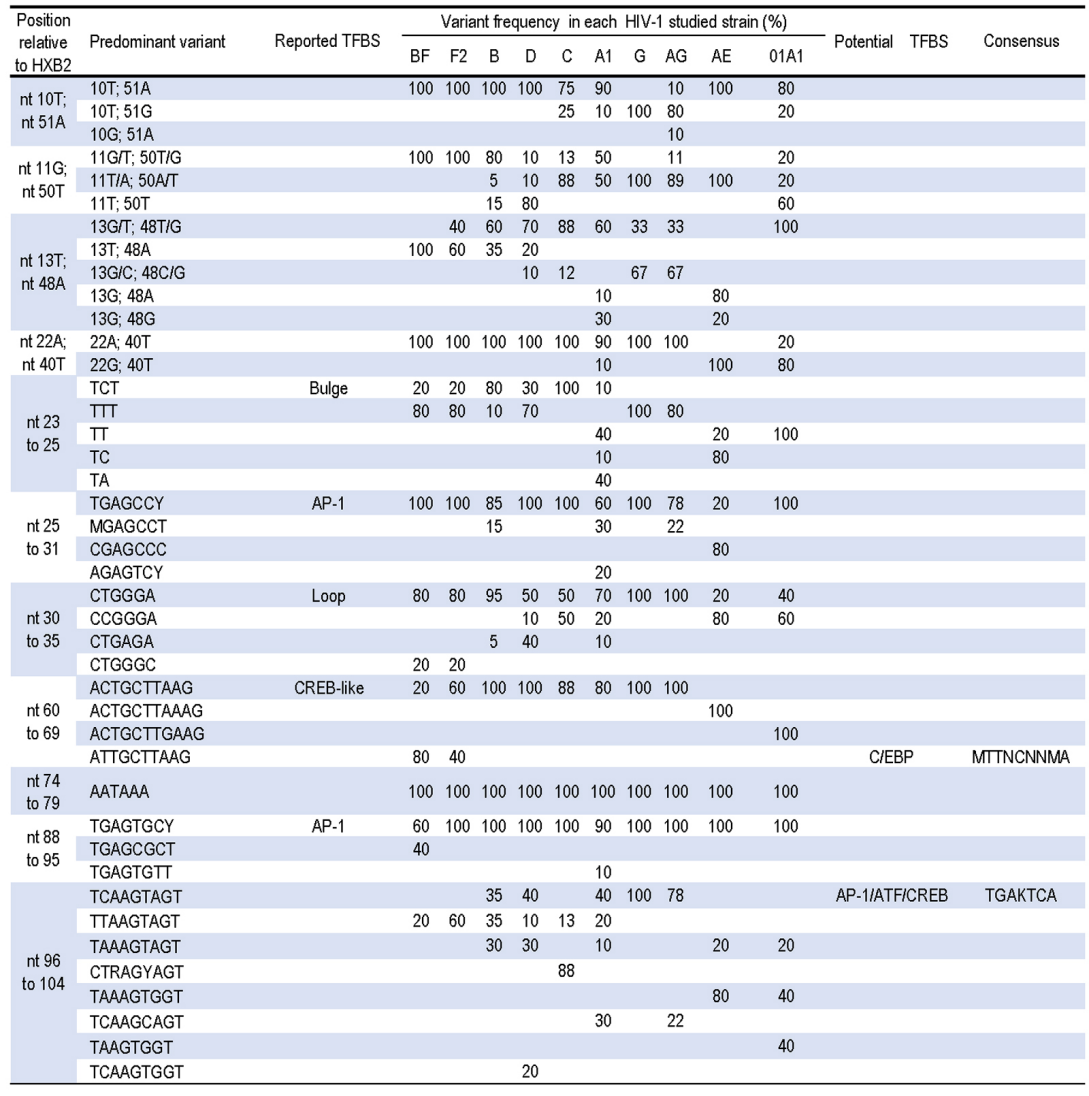
Variability in the R region of diverse HIV-1 subtypes and CRFs. N = any base; W = A or T; R = A or G; Y = C or T; K = G or T; M = A or C; S = G or C; D = A, G or T; H = A, C or T; B = C, G or T.

In accordance with previous reports, we found a C24T change in most of the CRF02_AG and majority of clades D, F2, and G isolates [39, 40], but also in CRF12_BF LTRs. Also, we found a deletion of T25 leading to a two nucleotides bulge in clade A1 and -CRF01_AE specimens as previously observed [5, 89], and in CRF22_01A1 as well (Fig 3). Furthermore, we noted that mutations of the bulge in clade A1 (60%) and CRF01_AE (80%) isolates mainly also affect the overlapping consensus TGAGCC(T/C) reported as a AP-1 recognition site [29]. The consensus motif of the loop was changed to a CCGGGA variant in most of the CR01_AE viruses as previously reported [89], and also in isolates CRF22_01A1 (60%), clades C (50%), and Al (20%). Additionally, a CTGAGAG variant of the loop was observed in clade D LTRs (40%) and in few sequences subtyped as A1 (10%) and B (5%). This latter variant of the loop converted the well conserved GGGAGCTCTC sequence reported as a NF-κB binding site [27], into a GAGAGCTCTC motif.

Mutations in the stem principally affect the A10:51T, G11:T50, T13:A48 and A22:T40 base pairing. We observed that most of the non-B strains displayed a cluster of point mutations rather than a single point mutation (Fig 3). Thus, while the single mutation A48G (affecting the T13:A48 pairing) was found in most of clade B LTRs, this change was associated with G11T clade D or with 11T22G in CRF22_01A1 specimens (Fig 3). In clade G and CRF02_AG specimens, we observed that the pairing T11:A50 and C13:G48 were associated with various other mutations especially in CRF02_AG viruses which displayed the highest variability for this feature of the TAR element. The T:A pairing at position 11 and 50 also found in CRF01_AE and clade C isolates, was associated with 13G and 13G48T co-variation respectively. The stem overlaps the footprint of a sequence reported to contain binding sites for cellular factors such as LBP-1 (nt -4 to +21) and CTF/NF-I [94, 95].

In the Poly-A hairpin (nt 61 to 105), the polyadenylation signal motif AATAAA (nt 74 to 79) as well as the AP-1 target (nt 88 to 95) [96, 97], were extremely well conserved in our study (Fig 3). We observed that mutation affecting the stem of the poly A hairpin lead to some strains specific patterns. Thus, the ACTGCTTAAG sequence (nt 60 to 69) which includes a CREB-like recognition site [98], was changed to ACTGCTTAAAG in CRF01_AE and to ACTGCTTGAAG in CRF22_01A1 specimens. In CRF12_BF (80%) and F2 (40%) LTRs, a C61T mutation transformed the CREB-like target ACTGCTTAA into an ATTGGCTTAA motif conformed to the canonical sequence for the binding of C/EBP proteins (Fig 3). The highest inter-clade variability of the poly A hairpin was observed within the HXB2 sequence TCAAGTAGT (nt 96 to 104) ending the R region. Indeed, this motif conserved in less than one-third of our analyzed sequences, was changed in a strains specific consensus such as CT(A/G)AG(C/T)AGT in clade C LTRs and TAA(A)iGTGGT is LTRs subtyped as CRF01_AE (Fig 3). The variant TTAAAGCAGT found in CRF12_BF and F2 LTRs includes a perfect match to the consensus binding site for CREB factors [98]. This potential TFBS may compensate the conversion of the reported CREB-like site (nt 60 to 69) into a potential C/EBP-like observed earlier in these LTR sequences.

## Discussion

To the best of our knowledge, this study is the first to describe the diversity of the 5’LTR U3R region from diverse HIV-1 strains including the newly emerging CRF22_01A1 HIV-1 strain. The CRF01_AE subtype assigned to sequences from CRF22_01A1 may be explained by the close relation of the two strains. Indeed, it has been reported that the gag, nef and segments of the env genes of a typical CRF22_01A1 strain, can be subtyped as CRF01_AE [47, 52]. The finding of additional contigs with different subtypes that are different from the primary genotype in some of our specimens can be explained as artefacts and/or contamination during our experiment. Alternatively, they can also result from the intra-host diversity of HIV-1 population. This highlights the fact that Next-generation sequencing, by detecting low-frequency mutation [42], might be a more reliable tool to analyze the genetic diversity of HIV-1. The intra-host heterogeneity of HIV-1 population reflects its high mutation rate and rapid turnover, but also the co-circulation of various HIV-1 strains within the same patient. Thus, the F2 contig found in a CRF02_AG specimen from Cameroon might corroborates the recent report of dual-infection with CRF02_AG and F2 in this country [99, 100]. Three out of five contigs assigned to be CRF12_BF were from clade B specimens collected in Bolivia, Spain and Japan. In these countries, a high prevalence or emerging BF recombinant circulating along with pure B have been reported [101–105].

The U3R region of the HIV-1 5’LTR is involved in the control of both basal and trans-activated transcription of the HIV-1 genome though its interaction with several cellular transcription factors. Therefore, mutations affecting its nucleotide sequence might drive potentials differences in the biological property of diverse HIV-1 strains. Here, we found that the degree of genetic variability of the U3R can vary from very low to fairly high, depending on the segment analyzed. Nevertheless, it has been demonstrated that even minor changes in this region could influence the replication rate and the disease severity of HIV-1 infection [15, 55].

In the U3 modulatory segment, besides binding sites for the RBE III and Ets-1 sites that were conserved in almost all the LTRs, majority of the TFBS were distinctive of one or few HIV-1 strains. Several motifs aligned with the TFBS reported in HXB2 displayed a sequence that varied from the reported consensus. As most of the regulatory elements that were studied belonged to clade B LTRs, functions of these TFBS described in non-B strains remains to be determined. Although the affinity for C/EBP proteins of the motifs found in non-B, C and D is yet to be investigated, it has been suggested that the binding ability and functions of C/EBP proteins can be modulated by interaction with other factors such as ATF/CREB/ AP-1 [106], E-boxes targeted proteins [107], and TATAA-binding proteins [108]. This might explain the finding of potential E-box (in CRF02_AG, clades B, C, D and G), AP-1 like (clade F2, CRF12_BF and CRF22_01A1) and Ets (CRF22_01A1 and CRF01_AE) binding sites in close proximity or embedded into the reported C/EBP binding site in our sequences analyzed. Thus, the differential repartition of these TFBS according to the HIV-1 strain might probably direct a distinctive mechanism of regulation of HIV-1 transcription. As previously reported, we also observed a high variability of the GATA binding motif. This variability has addressed the affinity, specificity and function of GATA factors [109]. Indeed, it has been reported that the specificity of GATA binding sites along with the spanning TFBS influence the fate of GATA-3 mediated T-cell differentiation and function [110]. Thus, the combination of strains-specific GATA binding sites along with distinctive spanning mutations may potentially impact pathogenicity of HIV-1 according to the strain.

Several TFBS have been shown to be able to activate or repress the transcriptional activity of HIV-1 depending on their affinity with the targeted factor and the spanning nucleotides [35, 71, 83, 84, 111–113]. Thus, in accordance with a recent report [35], the presence of the AP-1 variant TGACACA in CRF02_AG, CRF22_01A1, CRF12_BF, as well as in clades F2, C and A1 isolates may increase their capacity to establish HIV-1 latent infection compared to other strains. This predisposition may be affected by intra-clade variability due to the duplication of this variant within the MNFLP present in some of these specimens. On the other hand, the presence of an insert motif similar to the AP-1 activator of the p53 promoter might contribute to an increase reactivation of HIV-1 in F2 and CRF12_BF, as it has been shown that p53 could reactivate HIV-1 replication from its latency in U1 cells with upregulation/activation of host transcription factors such as AP-1 (Wang; X., et al, 2017, unpublished data).

In our study, the important TATA box element as well as key TFBS such as NF-κB was fairly well conserved. In accordance with previous reports [66, 114–116], the configuration and copy number of NF-κB sites found in our study; suggest inter-strain differences of the NF-κB induced activity. Also, as suggested by our finding in CRF02_AG specimens, this activity might also vary within isolates from the same HIV-1 strain. Furthermore, the variability found in the NF-κB inter-space required for the optimal binding of factors such as NFAT, Ets and AP-2 [87, 88, 117, 118], may also favor a distinctive modulation of the NF-κB activity in CRF01_AE, CRF22_01A1, CRF12_BF and clade F2. The 5T variant of the Sp III was shown to be able to abrogate the binding of Sp1 factors [90], and to be associated with HIV-1 disease progression [17]. As the mechanism leading to faster HIV-1 disease progression observed with CRF01_AE and some clade B isolates remain to be explored [114, 119–121], the contribution of Sp III along with other TFBS specificities should be considered. Also, it was reported that Sp1 and GABP factors cooperates to activate various genes such as CD18 which overexpression during HIV-1 infection has been related to disease severity [122]. The potential GABP binding site included in the Sp III variant of F2 and CRF12_BF LTRs, could also differentially impact the outcome of HIV-1 infection with these isolates.

In our study, the bulge residue T23, the loop as well as paired nucleotides below (A22:T40; G21:C41) and above (G26:C39 and A27:T38) shown to be important of the TAR activity were conserved in majority of the sequence analyzed. However, strain specific mutations within and outside these the features, might explained some differences in the transcriptional activity and replication rate between subtypes and CRFs [41, 66]. Changes in the Watson-Crick complementarity observed here, might also affect the TAR structure which has been shown to be important for its function, and for the activity of the downstream 5’LTR U5 region and the Gag leader sequence [123–125]. Also, it has been reported that regulation of the transcriptional activity driven by TAR-tat interaction can be enhanced through synergic action with various TFBS such the AP-1 targets with a crucial role in the replication and transcription of HIV-1 [97]. In most of the sequence analyzed in our study, the TFBS in the R region were well conserved assuming that the biological mechanism in which these TFBS are involved might be similar for various HIV-1 strains. Nevertheless, the potential loss of one of the AP-1/ATF/CREB-like sites in CRF01_AE and CRF22_01A1 specimens, and the relocation of the CREB-like in CRF12_BF and some F2 LTRs associated with the presence of a potential C/EBP site might suggest a distinctive TAR mediated activity for these isolates.

It is known that the differential activity of the LTR due to the lack or mutation of a TFBS can be compensated by various mechanisms such as the gain of additional factors, the interplay between TFBS in close proximity or the delocalization of the TFBS. On the other hand, association of potential strains distinctive mutations in the U3R may contribute to strain-specific function of the 5’LTRs [66, 116]. For CRF01_AE isolates for example, it was suggested that its 2nt bulge (TC), 22G31C and TAAAA co-mutations promote its higher transcriptional activity and replication rate compared with clade B [41, 66, 126].

In this study, we have reported distinguishing patterns within and outside the reported regulatory element in the U3R region from diverse HIV-1 strains. Due to the important role of this region in the control of the HIV-1 transcription, it is reasonable to suggest that strain specific variability greatly contributed to the effectiveness of replication and gene expression of different HIV-1 subtypes and CRFs. Intra and inter-clade variability of the U3R region should be further explored in future studies related to the replicative capacity and pathogenicity of HIV-1, as this may lead to a better understanding of phenotypic effects of HIV-1genetic diversity.

## Supporting information

S1 Table Genbank accession number of the 5’LTR sequence from diverse HIV-1 subtypes and CRFs. (XLSX)

## References

[1] Hemelaar J, Gouws E, Ghys PD, Osmanov S. C W-UNHI: global trends in molecular epidemiology of HIV-1 during 2000–2007. Aids. 2011;25 doi: 10.1097/QAD.0b013e328342ff93 21297424 10.1097/QAD.0b013e328342ff93 PMC3755761

[2] Pant Pai N, Shivkumar S, Cajas JM. Does Genetic Diversity of HIV-1 Non-B Subtypes Differentially Impact Disease Progression in Treatment-Naive HIV-1–Infected Individuals? A Systematic Review of Evidence: 1996–2010. JAIDS Journal of Acquired Immune Deficiency Syndromes. 2012;59(4):382–8. doi: 10.1097/QAI.0b013e31824a0628 -201204010-00010. 22269800 10.1097/QAI.0b013e31824a0628

[3] Taylor BS, Sobieszczyk ME, McCutchan FE, Hammer SM. The challenge of HIV-1 subtype diversity. N Engl J Med. 2008;358(15):1590–602. doi: 10.1056/NEJMra0706737 ; PubMed Central PMCID: PMC2614444. 18403767 10.1056/NEJMra0706737 PMC2614444

[4] Kanki PJ, Hamel DJ, Sankalé J-L, Hsieh C-c, Thior I, Barin F, et al Human Immunodeficiency Virus Type 1 Subtypes Differ in Disease Progression. The Journal of infectious diseases. 1999;179(1):68–73. doi: 10.1086/314557 9841824 10.1086/314557

[5] Rodriguez MA, Shen C, Ratner D, Paranjape RS, Kulkarni SS, Chatterjee R, et al Genetic and functional characterization of the LTR of HIV-1 subtypes A and C circulating in India. AIDS research and human retroviruses. 2007;23(11):1428–33. doi: 10.1089/aid.2007.0152 . 18184086 10.1089/aid.2007.0152

[6] Foy HM, Kunanusont C, Kreiss JK, Phanuphak P, Raktham S, Pau CP, et al HIV-1 subtypes and male-to-female transmission in Thailand. The Lancet. 1995;345(8957):1078–83. doi: 10.1016/S0140-6736(95)90818-8 10.1016/s0140-6736(95)90818-8 7715340

[7] Kiwanuka N, Laeyendecker O, Quinn TC, Wawer MJ, Shepherd J, Robb M, et al HIV-1 subtypes and differences in heterosexual HIV transmission among HIV-discordant couples in Rakai, Uganda. Aids. 2009;23(18):2479–84. doi: 10.1097/QAD.0b013e328330cc08 -200911270-00013. 19841572 10.1097/QAD.0b013e328330cc08 PMC2910931

[8] Baeten JM, Chohan B, Lavreys L, Chohan V, McClelland RS, Certain L, et al HIV-1 subtype D infection is associated with faster disease progression than subtype A in spite of similar plasma HIV-1 loads. The Journal of infectious diseases. 2007;195(8):1177–80. doi: 10.1086/512682 . 17357054 10.1086/512682

[9] Kiwanuka N, Robb M, Laeyendecker O, Kigozi G, Wabwire-Mangen F, Makumbi FE, et al HIV-1 viral subtype differences in the rate of CD4+ T-cell decline among HIV seroincident antiretroviral naive persons in Rakai district, Uganda. J Acquir Immune Defic Syndr. 2010;54(2):180–4. doi: 10.1097/QAI.0b013e3181c98fc0 ; PubMed Central PMCID: PMC2877752. 20010433 10.1097/QAI.0b013e3181c98fc0 PMC2877752

[10] Palm AA, Esbjornsson J, Mansson F, Biague A, da Silva ZJ, Norrgren H, et al Cocirculation of several similar but unique HIV-1 recombinant forms in Guinea-Bissau revealed by near full-length genomic sequencing. AIDS research and human retroviruses. 2015;31(9):938–45. doi: 10.1089/AID.2015.0073 . 26066756 10.1089/AID.2015.0073

[11] Beerens N, Klaver B, Berkhout B. A structured RNA motif is involved in correct placement of the tRNA(3)(Lys) primer onto the human immunodeficiency virus genome. Journal of virology. 2000;74(5):2227–38. ; PubMed Central PMCID: PMCPMC111704. 10666253 10.1128/jvi.74.5.2227-2238.2000 PMC111704

[12] Drosten C, Panning M, Drexler JF, Hänsel F, Pedroso C, Yeats J, et al Ultrasensitive Monitoring of HIV-1 Viral Load by a Low-Cost Real-Time Reverse Transcription-PCR Assay with Internal Control for the 5′ Long Terminal Repeat Domain. Clinical Chemistry. 2006;52(7):1258–66. doi: 10.1373/clinchem.2006.066498 16627558 10.1373/clinchem.2006.066498 PMC7108179

[13] Pereira LA, Bentley K, Peeters A, Churchill MJ, Deacon NJ. A compilation of cellular transcription factor interactions with the HIV-1 LTR promoter. Nucleic acids research. 2000;28(3):663–8. ; PubMed Central PMCID: PMC102541. 10637316 10.1093/nar/28.3.663 PMC102541

[14] van Opijnen T, Jeeninga RE, Boerlijst MC, Pollakis GP, Zetterberg V, Salminen M, et al Human immunodeficiency virus type 1 subtypes have a distinct long terminal repeat that determines the replication rate in a host-cell-specific manner. Journal of virology. 2004;78(7):3675–83. doi: 10.1128/JVI.78.7.3675-3683.2004 ; PubMed Central PMCID: PMC371093. 15016888 10.1128/JVI.78.7.3675-3683.2004 PMC371093

[15] Verhoef K, Sanders RW, Fontaine V, Kitajima S, Berkhout B. Evolution of the human immunodeficiency virus type 1 long terminal repeat promoter by conversion of an NF-kappaB enhancer element into a GABP binding site. Journal of virology. 1999;73(2):1331–40. ; PubMed Central PMCID: PMC103957. 9882338 10.1128/jvi.73.2.1331-1340.1999 PMC103957

[16] Burdo TH, Nonnemacher M, Irish BP, Choi CH, Krebs FC, Gartner S, et al High-affinity interaction between HIV-1 Vpr and specific sequences that span the C/EBP and adjacent NF-kappaB sites within the HIV-1 LTR correlate with HIV-1-associated dementia. DNA and cell biology. 2004;23(4):261–9. doi: 10.1089/104454904773819842 . 15142383 10.1089/104454904773819842

[17] Nonnemacher MR, Irish BP, Liu Y, Mauger D, Wigdahl B. Specific sequence configurations of HIV-1 LTR G/C box array result in altered recruitment of Sp isoforms and correlate with disease progression. Journal of neuroimmunology. 2004;157(1–2):39–47. doi: 10.1016/j.jneuroim.2004.08.021 . 15579278 10.1016/j.jneuroim.2004.08.021

[18] Gaynor R. Cellular transcription factors involved in the regulation of HIV-1 gene expression. Aids. 1992;6(4):347–63. . 1616633 10.1097/00002030-199204000-00001

[19] Ou S-HI, Gaynor RB. Intracellular Factors Involved in Gene Expression of Human Retroviruses In: Levy JA, editor. The Retroviridae. Boston, MA: Springer US; 1995 p. 97–184.

[20] Estable MC. In search of a function for the most frequent naturally-occurring length polymorphism (MFNLP) of the HIV-1 LTR: retaining functional coupling, of Nef and RBF-2, at RBEIII? International journal of biological sciences. 2007;3(5):318–27. ; PubMed Central PMCID: PMC1893116. 17589566 10.7150/ijbs.3.318 PMC1893116

[21] Estable MC, Bell B, Hirst M, Sadowski I. Naturally occurring human immunodeficiency virus type 1 long terminal repeats have a frequently observed duplication that binds RBF-2 and represses transcription. Journal of virology. 1998;72(8):6465–74. ; PubMed Central PMCID: PMC109809. 9658089 10.1128/jvi.72.8.6465-6474.1998 PMC109809

[22] Estable MC, Bell B, Merzouki A, Montaner JS, O'Shaughnessy MV, Sadowski IJ. Human immunodeficiency virus type 1 long terminal repeat variants from 42 patients representing all stages of infection display a wide range of sequence polymorphism and transcription activity. Journal of virology. 1996;70(6):4053–62. ; PubMed Central PMCID: PMC190286. 8648743 10.1128/jvi.70.6.4053-4062.1996 PMC190286

[23] Gaynor RB. Regulation of HIV-1 Gene Expression by the Transactivator Protein Tat In: Chen ISY, Koprowski H, Srinivasan A, Vogt PK, editors. Transacting Functions of Human Retroviruses. Berlin, Heidelberg: Springer Berlin Heidelberg; 1995 p. 51–77. 10.1007/978-3-642-78929-8_3 7648878

[24] Krebs FC, Hogan TH, Quiterio S, Gartner S, Wigdahl B. Lentiviral LTR-directed Expression, Sequence Variation, and Disease Pathogenesis In: Kuiken C, Foley B, Hahn B, Marx P, McCutchan F, Mellors JW, et al, editors. HIV Sequence Compendium 2001. Los Alamos, NM, LA-UR 02–2877.: Theoretical Biology and Biophysics Group, Los Alamos National Laboratory; 2001 p. 29–70

[25] Berkhout B, Klaver B, Das AT. A Conserved Hairpin Structure Predicted for the Poly(A) Signal of Human and Simian Immunodeficiency Viruses. Virology. 1995;207(1):276–81. doi: 10.1006/viro.1995.1077 7755727 10.1006/viro.1995.1077

[26] el Kharroubi A, Verdin E. Protein-DNA interactions within DNase I-hypersensitive sites located downstream of the HIV-1 promoter. Journal of Biological Chemistry. 1994;269(31):19916–24. 8051074

[27] Montano MA, Kripke K, Norina CD, Achacoso P, Herzenberg LA, Roy AL, et al NF-kB Homodimer Binding within the HIV-1 Initiator Region and Interactions with TFII-I. Proceedings of the National Academy of Sciences of the United States of America. 1996;93(22):12376–81. 8901589 10.1073/pnas.93.22.12376 PMC37999

[28] Ou SH, Wu F, Harrich D, García-Martínez LF, Gaynor RB. Cloning and characterization of a novel cellular protein, TDP-43, that binds to human immunodeficiency virus type 1 TAR DNA sequence motifs. Journal of virology. 1995;69(6):3584–96. 7745706 10.1128/jvi.69.6.3584-3596.1995 PMC189073

[29] Zoumpourlis V, Ergazaki M, Spandidos D. Ap-1 recognizes sequence elements on hiv-1 LTR in human epithelial tumor-cell lines. Oncology reports. 1994;1(2):397–401. . 21607373 10.3892/or.1.2.397

[30] Garcia JA, Harrich D, Soultanakis E, Wu F, Mitsuyasu R, Gaynor RB. Human immunodeficiency virus type 1 LTR TATA and TAR region sequences required for transcriptional regulation. The EMBO Journal. 1989;8(3):765–78. PubMed PMID: PMC400873. 2721501 10.1002/j.1460-2075.1989.tb03437.x PMC400873

[31] Karn J. Tackling tat. Journal of molecular biology. 1999;293(2):235–54. doi: 10.1006/jmbi.1999.3060 10550206 10.1006/jmbi.1999.3060

[32] Kilareski EM, Shah S, Nonnemacher MR, Wigdahl B. Regulation of HIV-1 transcription in cells of the monocyte-macrophage lineage. Retrovirology. 2009;6:118 doi: 10.1186/1742-4690-6-118 ; PubMed Central PMCID: PMC2805609. 20030845 10.1186/1742-4690-6-118 PMC2805609

[33] Kingsman SM, Kingsman AJ. The Regulation of Human Immunodeficiency Virus Type-1 Gene Expression. European Journal of Biochemistry. 1996;240(3):491–507. doi: 10.1111/j.1432-1033.1996.0491h.x 8856047 10.1111/j.1432-1033.1996.0491h.x

[34] Brigati C, Giacca M, Noonan DM, Albini A. HIV Tat, its TARgets and the control of viral gene expression. FEMS Microbiology Letters. 2003;220(1):57–65. doi: 10.1016/S0378-1097(03)00067-3 12644228 10.1016/S0378-1097(03)00067-3

[35] Duverger A, Wolschendorf F, Zhang M, Wagner F, Hatcher B, Jones J, et al An AP-1 binding site in the enhancer/core element of the HIV-1 promoter controls the ability of HIV-1 to establish latent infection. Journal of virology. 2013;87(4):2264–77. doi: 10.1128/JVI.01594-12 ; PubMed Central PMCID: PMC3571467. 23236059 10.1128/JVI.01594-12 PMC3571467

[36] Karn J, Stoltzfus CM. Transcriptional and Posttranscriptional Regulation of HIV-1 Gene Expression. Cold Spring Harbor Perspectives in Medicine. 2012;2(2):a006916 doi: 10.1101/cshperspect.a006916 PubMed PMID: PMC3281586. 22355797 10.1101/cshperspect.a006916 PMC3281586

[37] Terme J-M, Calvignac S, Duc Dodon M, Gazzolo L, Jordan A. E box motifs as mediators of proviral latency of human retroviruses. Retrovirology. 2009;6(1):81 doi: 10.1186/1742-4690-6-81 19758443 10.1186/1742-4690-6-81 PMC2749803

[38] Van Lint C, Bouchat S, Marcello A. HIV-1 transcription and latency: an update. Retrovirology. 2013;10(1):67 doi: 10.1186/1742-4690-10-67 23803414 10.1186/1742-4690-10-67 PMC3699421

[39] De Arellano ER, Soriano V, Holguin A. Genetic analysis of regulatory, promoter, and TAR regions of LTR sequences belonging to HIV type 1 Non-B subtypes. AIDS research and human retroviruses. 2005;21(11):949–54. doi: 10.1089/aid.2005.21.949 . 16386112 10.1089/aid.2005.21.949

[40] De Baar MP, De Ronde A, Berkhout B, Cornelissen M, Van Der Horn KH, Van Der Schoot AM, et al Subtype-specific sequence variation of the HIV type 1 long terminal repeat and primer-binding site. AIDS research and human retroviruses. 2000;16(5):499–504. doi: 10.1089/088922200309160 . 10772536 10.1089/088922200309160

[41] Jeeninga RE, Hoogenkamp M, Armand-Ugon M, de Baar M, Verhoef K, Berkhout B. Functional differences between the long terminal repeat transcriptional promoters of human immunodeficiency virus type 1 subtypes A through G. Journal of virology. 2000;74(8):3740–51. ; PubMed Central PMCID: PMC111883. 10729149 10.1128/jvi.74.8.3740-3751.2000 PMC111883

[42] Beerenwinkel N, Zagordi O. Ultra-deep sequencing for the analysis of viral populations. Curr Opin Virol. 2011;1(5):413–8. doi: 10.1016/j.coviro.2011.07.008 . 22440844 10.1016/j.coviro.2011.07.008

[43] Forns X, Bukh J, Purcell RH, Emerson SU. How Escherichia coli can bias the results of molecular cloning: preferential selection of defective genomes of hepatitis C virus during the cloning procedure. Proceedings of the National Academy of Sciences of the United States of America. 1997;94(25):13909–14. ; PubMed Central PMCID: PMC28406. 9391126 10.1073/pnas.94.25.13909 PMC28406

[44] Hora B, Keating SM, Chen Y, Sanchez AM, Sabino E, Hunt G, et al Genetic Characterization of a Panel of Diverse HIV-1 Isolates at Seven International Sites. PloS one. 2016;11(6):e0157340 doi: 10.1371/journal.pone.0157340 27314585 10.1371/journal.pone.0157340 PMC4912073

[45] Agyingi L, Mayr LM, Kinge T, Orock GE, Ngai J, Asaah B, et al The evolution of HIV-1 group M genetic variability in Southern Cameroon is characterized by several emerging recombinant forms of CRF02_AG and viruses with drug resistance mutations. Journal of medical virology. 2014;86(3):385–93. doi: 10.1002/jmv.23846 ; PubMed Central PMCID: PMC4011137. 24248638 10.1002/jmv.23846 PMC4011137

[46] Ragupathy V, Zhao J, Wood O, Tang S, Lee S, Nyambi P, et al Identification of new, emerging HIV-1 unique recombinant forms and drug resistant viruses circulating in Cameroon. Virology journal. 2011;8:185 doi: 10.1186/1743-422X-8-185 ; PubMed Central PMCID: PMC3118203. 21513545 10.1186/1743-422X-8-185 PMC3118203

[47] Zhao J, Tang S, Ragupathy V, Gaddam D, Wang X, Zhang P, et al CRF22_01A1 is involved in the emergence of new HIV-1 recombinants in Cameroon. J Acquir Immune Defic Syndr. 2012;60(4):344–50. Epub 2012/05/03. doi: 10.1097/QAI.0b013e318258c7e3 . 22549382 10.1097/QAI.0b013e318258c7e3 PMC3392544

[48] Sanchez AM, DeMarco CT, Hora B, Keinonen S, Chen Y, Brinkley C, et al Development of a contemporary globally diverse HIV viral panel by the EQAPOL program. J Immunol Methods. 2014;409:117–30. doi: 10.1016/j.jim.2014.01.004 ; PubMed Central PMCID: PMCPMC4104154. 24447533 10.1016/j.jim.2014.01.004 PMC4104154

[49] Kumar S, Stecher G, Tamura K. MEGA7: Molecular Evolutionary Genetics Analysis Version 7.0 for Bigger Datasets. Molecular biology and evolution. 2016;33(7):1870–4. doi: 10.1093/molbev/msw054 27004904 10.1093/molbev/msw054 PMC8210823

[50] Zhao J, Liu J, Vemula SV, Lin C, Tan J, Ragupathy V, et al Sensitive Detection and Simultaneous Discrimination of Influenza A and B Viruses in Nasopharyngeal Swabs in a Single Assay Using Next-Generation Sequencing-Based Diagnostics. PloS one. 2016;11(9):e0163175 doi: 10.1371/journal.pone.0163175 27658193 10.1371/journal.pone.0163175 PMC5033603

[51] Struck D, Lawyer G, Ternes A-MM, Schmit J-CC, Bercoff DP. COMET: adaptive context-based modeling for ultrafast HIV-1 subtype identification. Nucleic acids research. 2015;42. 10.1093/nar/gku739 PMC4191385 25120265

[52] Zhao J, Tang S, Ragupathy V, Carr JK, Wolfe ND, Awazi B, et al Identification and genetic characterization of a novel CRF22_01A1 recombinant form of HIV type 1 in Cameroon. AIDS research and human retroviruses. 2010;26(9):1033–45. Epub 2010/09/04. doi: 10.1089/aid.2009.0197 . 20812894 10.1089/aid.2009.0197 PMC2931544

[53] Schwartz C, Canonne-Hergaux F, Aunis D, Schaeffer E. Characterization of nuclear proteins that bind to the regulatory TGATTGGC motif in the human immunodeficiency virus type 1 long terminal repeat. Nucleic acids research. 1997;25(6):1177–84. ; PubMed Central PMCID: PMC146561. 9092627 10.1093/nar/25.6.1177 PMC146561

[54] Vemula SV, Veerasamy R, Ragupathy V, Biswas S, Devadas K, Hewlett I. H IV-1 Induced Nuclear Factor I-B (NF-IB) Expression Negatively Regulates HIV-1 Replication through Interaction with the Long Terminal Repeat Region. Viruses. 2015;7(2):543–58. doi: 10.3390/v7020543 PubMed PMID: PMC4353903. 25664610 10.3390/v7020543 PMC4353903

[55] Nonnemacher MR, Pirrone V, Feng R, Moldover B, Passic S, Aiamkitsumrit B, et al HIV-1 Promoter Single Nucleotide Polymorphisms Are Associated with Clinical Disease Severity. PloS one. 2016;11(4):e0150835 doi: 10.1371/journal.pone.0150835 PubMed PMID: PMC4839606. 27100290 10.1371/journal.pone.0150835 PMC4839606

[56] Van Lint C, Burny A, Verdin E. The intragenic enhancer of human immunodeficiency virus type 1 contains functional AP-1 binding sites. Journal of virology. 1991;65(12):7066–72. PubMed PMID: PMC250832. 1942259 10.1128/jvi.65.12.7066-7072.1991 PMC250832

[57] Franza BR Jr, Rauscher FJ 3rd, Josephs SF, Curran T. The Fos complex and Fos-related antigens recognize sequence elements that contain AP-1 binding sites. Science. 1988;239(4844):1150–3. . 2964084 10.1126/science.2964084

[58] Merika M, Orkin SH. DNA-binding specificity of GATA family transcription factors. Molecular and cellular biology. 1993;13(7):3999–4010. PubMed PMID: PMC359949. 8321207 10.1128/mcb.13.7.3999-4010.1993 PMC359949

[59] Yamamoto K, Mori S, Okamoto T, Shimotohno K, Kyogoku Y. Identification of transcriptional suppressor proteins that bind to the negative regulatory element of the human immunodeficiency virus type 1. Nucleic acids research. 1991;19(22):6107–12. ; PubMed Central PMCID: PMCPMC329097. 1956769 10.1093/nar/19.22.6107 PMC329097

[60] Pereira LA, Churchill MJ, Elefanty AG, Gouskos T, Lambert PF, Ramsay RG, et al Characterization of interactions between transcription factors and a regulatory region spanning nt -320 to -281 of the HIV-1 LTR in T-lymphoid and non-T-lymphoid cells. Journal of biomedical science. 2002;9(1):68–81. . 11810027 10.1007/BF02256580

[61] Yang Z, Engel JD. Human T cell transcription factor GATA-3 stimulates HIV-1 expression. Nucleic acids research. 1993;21(12):2831–6. PubMed PMID: PMC309663. 8332492 10.1093/nar/21.12.2831 PMC309663

[62] Georgakopoulos T, Aggeletopoulou I, Karagiannis F, Skoutelis A, Mouzaki A. Ets-2 Protein Is a Transcriptional Repressor Of The HIV-1 Virus and Acts Through Binding To The HIV-LTR-RATS Element. Blood. 2013;122(21):3477-.

[63] Wasylyk B, Hahn SL, Giovane A. The Ets family of transcription factors. European Journal of Biochemistry. 1993;211(1–2):7–18. doi: 10.1111/j.1432-1033.1993.tb19864.x 8425553 10.1007/978-3-642-78757-7_2

[64] Gustavson MD, Crawford HC, Fingleton B, Matrisian LM. Tcf binding sequence and position determines beta-catenin and Lef-1 responsiveness of MMP-7 promoters. Molecular carcinogenesis. 2004;41(3):125–39. doi: 10.1002/mc.20049 . 15457508 10.1002/mc.20049

[65] Markovitz DM, Hannibal MC, Smith MJ, Cossman R, Nabel GJ. Activation of the human immunodeficiency virus type 1 enhancer is not dependent on NFAT-1. Journal of virology. 1992;66(6):3961–5. ; PubMed Central PMCID: PMC241190. 1533884 10.1128/jvi.66.6.3961-3965.1992 PMC241190

[66] Montano MA, Novitsky VA, Blackard JT, Cho NL, Katzenstein DA, Essex M. Divergent transcriptional regulation among expanding human immunodeficiency virus type 1 subtypes. Journal of virology. 1997;71(11):8657–65. 9343223 10.1128/jvi.71.11.8657-8665.1997 PMC192329

[67] Argyropoulos C, Nikiforidis GC, Theodoropoulou M, Adamopoulos P, Boubali S, Georgakopoulos TN, et al Mining microarray data to identify transcription factors expressed in naive resting but not activated T lymphocytes. Genes Immun. 2004;5(1):16–25. doi: 10.1038/sj.gene.6364034 14735145 10.1038/sj.gene.6364034

[68] Cron RQ, Bartz SR, Clausell A, Bort SJ, Klebanoff SJ, Lewis DB. NFAT1 enhances HIV-1 gene expression in primary human CD4 T cells. Clinical immunology. 2000;94(3):179–91. doi: 10.1006/clim.1999.4831 . 10692237 10.1006/clim.1999.4831

[69] Churchill MJ, Ramsay RG, Rhodes DI, Deacon NJ. c-Myb Influences HIV Type 1 Gene Expression and Virus Production. AIDS research and human retroviruses. 2001;17(16):1481–8. doi: 10.1089/08892220152644188 11709092 10.1089/08892220152644188

[70] Mädge B. E-Box. In: Schwab M, editor. Encyclopedia of Cancer. Berlin, Heidelberg: Springer Berlin Heidelberg; 2009 p. 947–50.

[71] Ou SH, Garcia-Martínez LF, Paulssen EJ, Gaynor RB. Role of flanking E box motifs in human immunodeficiency virus type 1 TATA element function. Journal of virology. 1994;68(11):7188–99. PubMed PMID: PMC237158. 7933101 10.1128/jvi.68.11.7188-7199.1994 PMC237158

[72] Akira S, Isshiki H, Sugita T, Tanabe O, Kinoshita S, Nishio Y, et al A nuclear factor for IL-6 expression (NF-IL6) is a member of a C/EBP family. The EMBO Journal. 1990;9(6):1897–906. PubMed PMID: PMC551896. 2112087 10.1002/j.1460-2075.1990.tb08316.x PMC551896

[73] Ruocco MR, Chen X, Ambrosino C, Dragonetti E, Liu W, Mallardo M, et al Regulation of HIV-1 Long Terminal Repeats by Interaction of C/EBP(NF-IL6) and NF-κB/Rel Transcription Factors. Journal of Biological Chemistry. 1996;271(37):22479–86. doi: 10.1074/jbc.271.37.22479 8798413 10.1074/jbc.271.37.22479

[74] Tesmer VM, Rajadhyaksha A, Babin J, Bina M. NF-IL6-mediated transcriptional activation of the long terminal repeat of the human immunodeficiency virus type 1. Proceedings of the National Academy of Sciences of the United States of America. 1993;90(15):7298–302. PubMed PMID: PMC47124. 8346247 10.1073/pnas.90.15.7298 PMC47124

[75] Henderson AJ, Zou X, Calame KL. C/EBP proteins activate transcription from the human immunodeficiency virus type 1 long terminal repeat in macrophages/monocytes. Journal of virology. 1995;69(9):5337–44. PubMed PMID: PMC189374. 7636977 10.1128/jvi.69.9.5337-5344.1995 PMC189374

[76] Liu Y, Nonnemacher MR, Stauff DL, Li L, Banerjee A, Irish B, et al Structural and functional studies of CCAAT/enhancer binding sites within the human immunodeficiency virus type 1 subtype C LTR. Biomedicine & pharmacotherapy = Biomedecine & pharmacotherapie. 2010;64(10):672–80. doi: 10.1016/j.biopha.2010.09.007 ; PubMed Central PMCID: PMC2998390. 20970301 10.1016/j.biopha.2010.09.007 PMC2998390

[77] Estable MC, Hirst M, Bell B, O'Shaughnessy MV, Sadowski I. Purification of RBF-2, a transcription factor with specificity for the most conserved cis-element of naturally occurring HIV-1 LTRs. Journal of biomedical science. 1999;6(5):320–32. doi: 25404. doi: 10.1159/000025404 . 10494039 10.1007/BF02253521

[78] Holzmeister J, Ludewig B, Pauli G, Simon D. Sequence-Specific Binding of the Transcription Factor c-Ets1 to the Human Immunodeficiency Virus Type I Long Terminal Repeat. Biochemical and Biophysical Research Communications. 1993;197(3):1229–33. https://doi.org/10.1006/bbrc.1993.2608. doi: 10.1006/bbrc.1993.2608 8280137 10.1006/bbrc.1993.2608

[79] Mondal D, Alam J, Prakash O. NF-kappa B site-mediated negative regulation of the HIV-1 promoter by CCAAT/enhancer binding proteins in brain-derived cells. Journal of molecular neuroscience: MN. 1994;5(4):241–58. doi: 10.1007/BF02736725 . 7577367 10.1007/BF02736725

[80] Dahiya S, Liu Y, Nonnemacher MR, Dampier W, Wigdahl B. CCAAT enhancer binding protein and nuclear factor of activated T cells regulate HIV-1 LTR via a novel conserved downstream site in cells of the monocyte-macrophage lineage. PloS one. 2014;9(2):e88116 doi: 10.1371/journal.pone.0088116 ; PubMed Central PMCID: PMC3925103. 24551078 10.1371/journal.pone.0088116 PMC3925103

[81] Naghavi MH, Schwartz S, Sonnerborg A, Vahlne A. Long terminal repeat promoter/enhancer activity of different subtypes of HIV type 1. AIDS research and human retroviruses. 1999;15(14):1293–303. doi: 10.1089/088922299310197 . 10505678 10.1089/088922299310197

[82] Sheridan PL, Sheline CT, Cannon K, Voz ML, Pazin MJ, Kadonaga JT, et al Activation of the HIV-1 enhancer by the LEF-1 HMG protein on nucleosome-assembled DNA in vitro. Genes Dev. 1995;9(17):2090–104. . 7657162 10.1101/gad.9.17.2090

[83] d'Adda di Fagagna F, Marzio G, Gutierrez MI, Kang LY, Falaschi A, Giacca M. Molecular and functional interactions of transcription factor USF with the long terminal repeat of human immunodeficiency virus type 1. Journal of virology. 1995;69(5):2765–75. 7707499 10.1128/jvi.69.5.2765-2775.1995 PMC188970

[84] Naghavi MH, Estable MC, Schwartz S, Roeder RG, Vahlne A. Upstream stimulating factor affects human immunodeficiency virus type 1 (HIV-1) long terminal repeat-directed transcription in a cell-specific manner, independently of the HIV-1 subtype and the core-negative regulatory element. The Journal of general virology. 2001;82(Pt 3):547–59. doi: 10.1099/0022-1317-82-3-547 . 11172096 10.1099/0022-1317-82-3-547

[85] Kirch HC, Flaswinkel S, Rumpf H, Brockmann D, Esche H. Expression of human p53 requires synergistic activation of transcription from the p53 promoter by AP-1, NF-kappaB and Myc/Max. Oncogene. 1999;18(17):2728–38. doi: 10.1038/sj.onc.1202626 . 10348347 10.1038/sj.onc.1202626

[86] Bachu M, Yalla S, Asokan M, Verma A, Neogi U, Sharma S, et al Multiple NF-kappaB sites in HIV-1 subtype C long terminal repeat confer superior magnitude of transcription and thereby the enhanced viral predominance. The Journal of biological chemistry. 2012;287(53):44714–35. doi: 10.1074/jbc.M112.397158 ; PubMed Central PMCID: PMC3531786. 23132857 10.1074/jbc.M112.397158 PMC3531786

[87] Bassuk AG, Anandappa RT, Leiden JM. Physical interactions between Ets and NF-kappaB/NFAT proteins play an important role in their cooperative activation of the human immunodeficiency virus enhancer in T cells. Journal of virology. 1997;71(5):3563–73. ; PubMed Central PMCID: PMC191503. 9094628 10.1128/jvi.71.5.3563-3573.1997 PMC191503

[88] Perkins ND, Agranoff AB, Duckett CS, Nabel GJ. Transcription factor AP-2 regulates human immunodeficiency virus type 1 gene expression. Journal of virology. 1994;68(10):6820–3. ; PubMed Central PMCID: PMC237111. 8084021 10.1128/jvi.68.10.6820-6823.1994 PMC237111

[89] Kurosu T, Mukai T, Auwanit W, Ayuthaya PI, Saeng-Aroon S, Ikuta K. Variable sequences in the long terminal repeat and Its downstream region of some of HIV Type 1 CRF01_AE recently distributing among Thai carriers. AIDS research and human retroviruses. 2001;17(9):863–6. doi: 10.1089/088922201750252061 . 11429128 10.1089/088922201750252061

[90] McAllister JJ, Phillips D, Millhouse S, Conner J, Hogan T, Ross HL, et al Analysis of the HIV-1 LTR NF-kappaB-proximal Sp site III: evidence for cell type-specific gene regulation and viral replication. Virology. 2000;274(2):262–77. doi: 10.1006/viro.2000.0476 . 10964770 10.1006/viro.2000.0476

[91] Shah S, Alexaki A, Pirrone V, Dahiya S, Nonnemacher MR, Wigdahl B. Functional properties of the HIV-1 long terminal repeat containing single-nucleotide polymorphisms in Sp site III and CCAAT/enhancer binding protein site I. Virology journal. 2014;11:92 doi: 10.1186/1743-422X-11-92 ; PubMed Central PMCID: PMC4047001. 24886416 10.1186/1743-422X-11-92 PMC4047001

[92] Jones K, Kadonaga J, Luciw P, Tjian R. Activation of the AIDS retrovirus promoter by the cellular transcription factor, Sp1. Science. 1986;232(4751):755–9. doi: 10.1126/science.3008338 3008338 10.1126/science.3008338

[93] Liu Y-Z, Latchman DS. The octamer-binding proteins Oct-1 and Oct-2 repress the HIV long terminal repeat promoter and its transactivation by Tat. Biochemical Journal. 1997;322(1):155. 9078256 10.1042/bj3220155 PMC1218171

[94] Jones KA, Luciw PA, Duchange N. Structural arrangements of transcription control domains within the 5'-untranslated leader regions of the HIV-1 and HIV-2 promoters. Genes & Development. 1988;2(9):1101–14. doi: 10.1101/gad.2.9.1101 10.1101/gad.2.9.1101 2847959

[95] Moses AV, Ibanez C, Gaynor R, Ghazal P, Nelson JA. Differential role of long terminal repeat control elements for the regulation of basal and Tat-mediated transcription of the human immunodeficiency virus in stimulated and unstimulated primary human macrophages. Journal of virology. 1994;68(1):298–307. PubMed PMID: PMC236289. 8254741 10.1128/jvi.68.1.298-307.1994 PMC236289

[96] Roebuck KA, Brenner DA, Kagnoff MF. Identification of c-fos-responsive elements downstream of TAR in the long terminal repeat of human immunodeficiency virus type-1. Journal of Clinical Investigation. 1993;92(3):1336–48. PubMed PMID: PMC288275. doi: 10.1172/JCI116707 8376588 10.1172/JCI116707 PMC288275

[97] Van Lint C, Amella CA, Emiliani S, John M, Jie T, Verdin E. Transcription factor binding sites downstream of the human immunodeficiency virus type 1 transcription start site are important for virus infectivity. Journal of virology. 1997;71(8):6113–27. ; PubMed Central PMCID: PMC191872. 9223506 10.1128/jvi.71.8.6113-6127.1997 PMC191872

[98] Sassone-Corsi P. Transcription factors responsive to cAMP. Annu Rev Cell Dev Biol. 1995;11:355–77. doi: 10.1146/annurev.cb.11.110195.002035 . 8689562 10.1146/annurev.cb.11.110195.002035

[99] Courtney CR, Agyingi L, Fokou A, Christie S, Asaah B, Meli J, et al Monitoring HIV-1 Group M Subtypes in Yaoundé, Cameroon Reveals Broad Genetic Diversity and a Novel CRF02_AG/F2 Infection. AIDS research and human retroviruses. 2015;32(4):381–5. doi: 10.1089/aid.2015.0286 26681241 10.1089/aid.2015.0286 PMC4817561

[100] Powell RLR, Urbanski MM, Burda S, Kinge T, Nyambi PN. High Frequency of HIV-1 Dual Infections Among HIV-Positive Individuals in Cameroon, West Central Africa. JAIDS Journal of Acquired Immune Deficiency Syndromes. 2009;50(1):84–92. doi: 10.1097/QAI.0b013e31818d5a40 . 19295338 10.1097/QAI.0b013e31818d5a40

[101] González-Alba JM, Holguín Á, Garcia R, García-Bujalance S, Alonso R, Suárez A, et al Molecular Surveillance of HIV-1 in Madrid, Spain: a Phylogeographic Analysis. Journal of virology. 2011;85(20):10755–63. doi: 10.1128/JVI.00454-11 PubMed PMID: PMC3187488. 21795343 10.1128/JVI.00454-11 PMC3187488

[102] Guimarães ML, Velarde-Dunois KG, Segurondo D, Morgado MG. The HIV-1 epidemic in Bolivia is dominated by subtype B and CRF12_BF "family" strains. Virology journal. 2012;9:19-. doi: 10.1186/1743-422X-9-19 PubMed PMID: PMC3285048. 22248191 10.1186/1743-422X-9-19 PMC3285048

[103] Leal É, Villanova FE. Diversity of HIV-1 Subtype B: Implications to the Origin of BF Recombinants. PloS one. 2010;5(7):e11833 doi: 10.1371/journal.pone.0011833 20676362 10.1371/journal.pone.0011833 PMC2911370

[104] Shiino T, Sadamasu K, Hattori J, Nagashima M, Iwatani Y, Yokomaku Y, et al. Large MSM Group and Local Heterosexual Transmission Are Major Concerns in the HIV Epidemic in Japan. Conference on Retroviruses and Opportunistic Infections (CROI); March 3–6, 2014 Boston, Massachusetts2014.

[105] Sierra M, Thomson MM, Ríos M, Casado G, Castro RO-d, Delgado E, et al The analysis of near full-length genome sequences of human immunodeficiency virus type 1 BF intersubtype recombinant viruses from Chile, Venezuela and Spain reveals their relationship to diverse lineages of recombinant viruses related to CRF12_BF. Infection, Genetics and Evolution. 2005;5(3):209–17. doi: 10.1016/j.meegid.2004.07.010 15737911 10.1016/j.meegid.2004.07.010

[106] Ross HL, Nonnemacher MR, Hogan TH, Quiterio SJ, Henderson A, McAllister JJ, et al Interaction between CCAAT/Enhancer Binding Protein and Cyclic AMP Response Element Binding Protein 1 Regulates Human Immunodeficiency Virus Type 1 Transcription in Cells of the Monocyte/Macrophage Lineage. Journal of virology. 2001;75(4):1842–56. doi: 10.1128/JVI.75.4.1842-1856.2001 PubMed PMID: PMC114094. 11160683 10.1128/JVI.75.4.1842-1856.2001 PMC114094

[107] Schwartz C, Catez P, Rohr O, Lecestre D, Aunis D, Schaeffer E. Functional interactions between C/EBP, Sp1, and COUP-TF regulate human immunodeficiency virus type 1 gene transcription in human brain cells. Journal of virology. 2000;74(1):65–73. ; PubMed Central PMCID: PMC111514. 10590092 10.1128/jvi.74.1.65-73.2000 PMC111514

[108] Liu Y, Nonnemacher MR, Wigdahl B. CCAAT/enhancer-binding proteins and the pathogenesis of retrovirus infection. Future microbiology. 2009;4:299–321. doi: 10.2217/fmb.09.4 PubMed PMID: PMC2710768. 19327116 10.2217/fmb.09.4 PMC2710768

[109] Bresnick EH, Katsumura KR, Lee H-Y, Johnson KD, Perkins AS. Master regulatory GATA transcription factors: mechanistic principles and emerging links to hematologic malignancies. Nucleic acids research. 2012;40(13):5819–31. doi: 10.1093/nar/gks281 22492510 10.1093/nar/gks281 PMC3401466

[110] Wei G, Abraham BJ, Yagi R, Jothi R, Cui K, Sharma S, et al Genome-wide analyses of transcription factor GATA3-mediated gene regulation in distinct T cell types. Immunity. 2011;35(2):299–311. doi: 10.1016/j.immuni.2011.08.007 PubMed PMID: PMC3169184. 21867929 10.1016/j.immuni.2011.08.007 PMC3169184

[111] Colin L, Vandenhoudt N, de Walque S, Van Driessche B, Bergamaschi A, Martinelli V, et al The AP-1 Binding Sites Located in the pol Gene Intragenic Regulatory Region of HIV-1 Are Important for Viral Replication. PloS one. 2011;6(4):e19084 doi: 10.1371/journal.pone.0019084 PubMed PMID: PMC3079759. 21526160 10.1371/journal.pone.0019084 PMC3079759

[112] Imai K, Okamoto T. Transcriptional repression of human immunodeficiency virus type 1 by AP-4. The Journal of biological chemistry. 2006;281(18):12495–505. doi: 10.1074/jbc.M511773200 . 16540471 10.1074/jbc.M511773200

[113] Stojanova A, Caro C, Jarjour RJV, Oster SK, Penn LZ, Germinario RJ. Repression of the human immunodeficiency virus type-1 long terminal repeat by the c-Myc oncoprotein. Journal of Cellular Biochemistry. 2004;92(2):400–13. doi: 10.1002/jcb.20065 15108364 10.1002/jcb.20065

[114] Qu D, Li C, Sang F, Li Q, Jiang Z-Q, Xu L-R, et al The variances of Sp1 and NF-κB elements correlate with the greater capacity of Chinese HIV-1 B′-LTR for driving gene expression. Scientific Reports. 2016;6:34532 doi: 10.1038/srep34532 PubMed PMID: PMC5048295. 27698388 10.1038/srep34532 PMC5048295

[115] Kurosu T, Mukai T, Komoto S, Ibrahim MS, Li Y-g, Kobayashi T, et al Human Immunodeficiency Virus Type 1 Subtype C Exhibits Higher Transactivation Activity of Tat than Subtypes B and E. Microbiology and Immunology. 2002;46(11):787–99. doi: 10.1111/j.1348-0421.2002.tb02766.x 12516777 10.1111/j.1348-0421.2002.tb02766.x

[116] Verma A, Rajagopalan P, Lotke R, Varghese R, Selvam D, Kundu TK, et al Functional Incompatibility between the Generic NF-κB Motif and a Subtype-Specific Sp1III Element Drives the Formation of the HIV-1 Subtype C Viral Promoter. Journal of virology. 2016;90(16):7046–65. doi: 10.1128/JVI.00308-16 27194770 10.1128/JVI.00308-16 PMC4984642

[117] Kinoshita S, Su L, Amano M, Timmerman LA, Kaneshima H, Nolan GP. The T cell activation factor NF-ATc positively regulates HIV-1 replication and gene expression in T cells. Immunity. 1997;6(3):235–44. . 9075924 10.1016/s1074-7613(00)80326-x

[118] Romanchikova N, Ivanova V, Scheller C, Jankevics E, Jassoy C, Serfling E. NFAT transcription factors control HIV-1 expression through a binding site downstream of TAR region. Immunobiology. 2003;208(4):361–5. doi: 10.1078/0171-2985-00283 . 14748509 10.1078/0171-2985-00283

[119] Keller M, Lu Y, Lalonde RG, Klein MB. Impact of HIV-1 viral subtype on CD4+ T-cell decline and clinical outcomes in antiretroviral naive patients receiving universal healthcare. Aids. 2009;23(6):731–7. doi: 10.1097/QAD.0b013e328326f77f . 19279446 10.1097/QAD.0b013e328326f77f

[120] Li Y, Han Y, Xie J, Gu L, Li W, Wang H, et al CRF01_AE subtype is associated with X4 tropism and fast HIV progression in Chinese patients infected through sexual transmission. Aids. 2014;28(4):521–30. doi: 10.1097/QAD.0000000000000125 . 24472744 10.1097/QAD.0000000000000125

[121] Rangsin R, Piyaraj P, Sirisanthana T, Sirisopana N, Short O, Nelson KE. The natural history of HIV-1 subtype E infection in young men in Thailand with up to 14 years of follow-up. Aids. 2007;21:S39–S46. doi: 10.1097/01.aids.0000299409.29528.23 . 10.1097/01.aids.0000299409.29528.23 18032937

[122] Palmer S, Hamblin AS. Increased CD11/CD18 expression on the peripheral blood leucocytes of patients with HIV disease: relationship to disease severity. Clinical and experimental immunology. 1993;93(3):344–9. PubMed PMID: PMC1554900. 8103716 10.1111/j.1365-2249.1993.tb08183.x PMC1554900

[123] Beerens N, Groot F, Berkhout B. Stabilization of the U5-leader stem in the HIV-1 RNA genome affects initiation and elongation of reverse transcription. Nucleic acids research. 2000;28(21):4130–7. ; PubMed Central PMCID: PMCPMC113157. 11058109 10.1093/nar/28.21.4130 PMC113157

[124] Vrolijk MM, Ooms M, Harwig A, Das AT, Berkhout B. Destabilization of the TAR hairpin affects the structure and function of the HIV-1 leader RNA. Nucleic acids research. 2008;36(13):4352–63. doi: 10.1093/nar/gkn364 ; PubMed Central PMCID: PMC2490758. 18586822 10.1093/nar/gkn364 PMC2490758

[125] Berkhout B. Structure and Function of the Human Immunodeficiency Virus Leader RNA. Progress in Nucleic Acid Research and Molecular Biology. 1996;54:1–34. https://doi.org/10.1016/S0079-6603(08)60359-1. 8768071 10.1016/s0079-6603(08)60359-1

[126] Kozaczynska K, Cornelissen M, Reiss P, Zorgdrager F, van der Kuyl AC. HIV-1 sequence evolution in vivo after superinfection with three viral strains. Retrovirology. 2007;4(1):59 doi: 10.1186/1742-4690-4-59 17716368 10.1186/1742-4690-4-59 PMC2020475

